# c-Myc Targets HDAC3 to Suppress NKG2DL Expression and Innate Immune Response in N-Type SCLC through Histone Deacetylation

**DOI:** 10.3390/cancers14030457

**Published:** 2022-01-18

**Authors:** Peiyan Zhao, Xiaodan Sun, Hui Li, Yan Liu, Yanan Cui, Lin Tian, Ying Cheng

**Affiliations:** 1 Postdoctoral Research Workstation, Jilin Cancer Hospital, Changchun 130012, China; zhaopy18@163.com (P.Z.); 15640584861@163.com (X.S.); 2 Medical Oncology Translational Research Lab, Jilin Cancer Hospital, Changchun 130012, China; lihui181966963@163.com (H.L.); shishui11245@163.com (Y.L.); tianlin602@163.com (L.T.); 3 Department of Thoracic Oncology, Jilin Cancer Hospital, Changchun 130012, China; cuiyanan9257@163.com

**Keywords:** SCLC, c-Myc, NKG2DL, HDAC3, innate immune response, histone deacetylation

## Abstract

**Simple Summary:**

Natural killer group 2, member D ligand (NKG2DL) is the most relevant ligand of NK cells to perform immune surveillance and is rarely expressed in most small cell lung cancer (SCLC) with the unclear mechanism. This study aimed to investigate the mechanisms underlying the NKG2DL deficiency in *C-MYC* (*MYC*)-amplificated N-type SCLC (SCLC-N) with less immune infiltrate. Our data showed that c-Myc was the suppressor of NKG2DL in SCLC-N. Further, c-Myc suppressed the transcription of NKG2DL by recruiting HDAC3 to deacetylate H3K9ac at the promoter of *MICA* and *MICB* in SCLC-N and inhibited the cytotoxicity of NK cells. The above findings revealed the role of c-Myc/HDAC3 axis in the regulation of NKG2DL expression, supplying a new perception for comprehending the mechanism of SCLC-N immune escape, which was poorly understood and providing the therapeutic targets that SCLC-N may benefit from.

**Abstract:**

SCLC is an aggressive malignancy with a very poor prognosis and limited effective therapeutic options. Despite the high tumor mutational burden, responses to immunotherapy are rare in SCLC patients, which may be due to the lack of immune surveillance. Here, we aimed to examine the role and mechanism of oncogene *MYC* in the regulation of NKG2DL, the most relevant NK-activating ligand in SCLC-N. Western Blotting, Immunofluorescence, flow cytometry, quantitative real-time PCR (qRT-PCR), Co-Immunoprecipitation (Co-IP), chromatin immunoprecipitation (ChIP), and Cytotoxicity assay were used on H2227 cells, H446 cells, and other SCLC cell lines, and we found that c-Myc negatively regulated NKG2DL expression in SCLC-N cells. Mechanistically, c-Myc recruited HDAC3 to deacetylate H3K9ac at the promoter regions of *MICA* and *MICB*, suppressing the MICA/B expression of SCLC-N cells and the cytotoxicity of NK cells. Treatment with selective HDAC3 inhibitor up-regulated the expression of NKG2DL on SCLC-N cells and increased the cytotoxicity of NK cells. Furthermore, analysis of the CCLE and Kaplan-Meier plotter data performed the negative correlation between *MYC* and *NKG2DL* in SCLC-N cells and the correlation with the prognosis of lung cancer patients. Collectively, the results provided the new insight into the role and mechanism of c-Myc/HDAC3 axis in NKG2DL expression and innate immune escape of SCLC-N, suggesting the potential target for SCLC-N immunotherapy.

## 1. Introduction

Small cell lung cancer (SCLC), an extremely malignant subtype of lung cancer, is featured by rapid growth and tendency to metastasize, with a dismal prognosis and high relapse rate. Among all patients with SCLC, about two-thirds are diagnosed with advanced-stage disease that is generally accompanied by distant metastasis and has a five-year survival rate of less than 5% [1]. SCLC is sensitive to chemoradiotherapy, but most patients experience disease relapse and drug resistance within six months [2]. In the immunotherapy era, despite the addition of immunotherapy to frontline chemotherapy, the absolute improvements in progression-free survival (PFS) and overall survival (OS) are modest and the effective rate of immune checkpoint inhibitor monotherapy is only 10–20% [3,4,5,6]. Therefore, there is an urgent need to identify new targets or treatment modalities to improve the effectiveness of immunotherapy for SCLC. Abundant clinical trials of immunotherapy that do not differentiate among SCLC patients are underway, but the results of the experiments are disappointing.

In SCLC, responses to immune checkpoint blockade (ICB) are rare, which may be related with the low infiltration by immune cells, especially cytotoxic lymphocytes [7,8]. SCLC has an immune cold tumor microenvironment (TME) with few infiltrated cytotoxic lymphocytes and one-fifth total immune cells compared with NSCLC [9]. Antitumor activity of the immune system largely depends on cytotoxic cells, T cells, and NK cells. While T cells depend on specific antigens and act as an important component of adaptive immune response, NK cells are part of innate immunity and recognize tumors by germline-encoded patterns [10]. NK cells are critical in preventing lung tumor growth, as depletion of NK cells were shown to facilitate lung cancer initiation and metastasis in experimental models [11,12]. They are activated by natural killer group 2, member D ligand (NKG2DL) present on the surface of tumor cells and attack tumor cells by secreting cytotoxic proteins, such as perforin and granzymes [13,14]. In humans, NKG2DL can be classified into two subsets, MICA/B and ULBP. While mice only have orthologs of human *RAET1* genes, including *Rae1α/β/γ/δ/ε*, *MULT1*, and *H60a/b/c* [15]. NKG2D is expressed on cytotoxic T cells, and NKG2DL can stimulate cytotoxicity of T cells [16]. Our previous research found that NKG2DL could recruit the cytotoxicity immune cells into the tumor nest [17]. However, according to reports, SCLC cell lines and patient-derived SCLC showed a significantly lower level of total NKG2DL compared with NSCLC cell lines [18], suggesting that NKG2DL might be the key molecule that modulates the immunosuppressive TME of SCLC.

SCLC has been treated as a single disease without patient stratification and exhibits genetic loss of both tumor suppressors *RB1* and *TP53*, along with mutually exclusive expression of *MYC* paralog (*MYC*, *MYCN*, *MYCL*) [19,20,21,22,23]. Nowadays, next-generation gene sequencing-based gene expression analysis of human tumors and cell lines revealed that SCLC comprised four distinct subtypes based on the expression of ASCL1(SCLC-A), NeuroD1(SCLC-N), YAP1(SCLC-Y), and POU2F3(SCLC-P) [24]. The research on SCLC typing is endless, and the typing methods are not completely consistent. The researchers analyzed the sequencing results of patients enrolled in the IMpower133 study and named the ASCL1^−^NeuroD1^−^POU2F3^−^ tumor tissues as subtype-I (SCLC-I) with high expression of inflammatory genes and increased number of immune cells including T cells, NK cells, and macrophages [8]. Survival analysis showed that SCLC-I tumors derived greater benefit from ICB. According to the reports that *MYC* drove the SCLC-N and SCLC-Y subtype of SCLC in a temporal evolution by reprograming neuroendocrine fate [25], SCLC-N cell lines highly expressed c-Myc with low level of NKG2DL and showed less immune infiltrate than other types [26], and SCLC-Y cell lines expressed low level of c-Myc with high level of NKG2DL [8], we speculated that *MYC* may contribute to the construction of SCLC-N immune microenvironment and the effect of immunotherapy through modulating NKG2DL.

Here, the study aimed to identify the c-Myc-dependent mechanism of suppressing NKG2DL expression and innate immune response in SCLC-N and found that c-Myc targeted HDAC3 to deacetylate H3K9ac at *NKG2DL* promoter and inhibited the transcription of *NKG2DL* in SCLC-N cells. The study might suggest the inhibition of HDAC3 to be the selectively beneficial therapeutic approaches for *MYC*-amplificated SCLC-N.

## 2. Materials and Methods

### 2.1. Cell Culture

Human SCLC cell lines NCI-H2227 (H2227), NCI-H446 (H446), NCI-H69 (H69), NCI-H524 (H524), and NCI-H196 (H196) (American Type Culture Collection, Manassas, VA, USA) were maintained at 37 °C in RPMI-1640 medium or Dulbecco’s Modified Eagle Media supplemented with 10% (*v*/*v*) FBS and antibiotics (100 IU/mL of penicillin and streptomycin). Human malignant non-Hodgkins Lymphoma cell line NK-92MI were maintained at 37 °C in NK-92MI special complete medium (Procell, Wuhan, China). All the cell lines were cultured in a 5% CO_2_ in air humidified incubator.

### 2.2. Plasmids and Transfection

For the overexpression of c-Myc, full-length *MYC* encoding sequence (CDS) (Gene ID: 4609) was subcloned into CMV-MCS-IRES-EGFP-SV40-Neomycin vector (MYC-OE, GENECHEM, Shanghai, China). The empty vector was used as a negative control. All plasmids were isolated using TIANprep Mini Plasmid Kit (DP103, TIANGEN, Beijing, China) and transfected using Lipofectamine 3000 Reagent (Thermo Fisher Scientific, Waltham, MA, USA) according to the manufacture’s protocol. After 48 or 72 h, the cells were collected for the following studies.

### 2.3. MYC siRNA Transfection

Endogenous c-Myc expression in H446 cells and H2227 cells was reduced using transient siRNA transfection. Around 24 h post-seeding when reaching 50% cell density, the cells were transfected with either 40 nM of *MYC* siRNAs (siMYC#1, GAGGAUAUCUGGAAGAAAUTT; siMYC#2, GCUUGUACCUGCAGGAUCUTT; siMYC#3, GGAAGAAAUCGAUGUUGUUTT) or 40 nM of Negative Control siRNA (NC-siRNA, UUCUCCGAACGUGUCACGUTT) (GeneParma, Shanghai, China). The transfection was performed for 4 h using a dilution of 1:50 in Lipofectamine 2000 (Thermo Fisher Scientific, Waltham, MA, USA) in serum-free DMEM followed by a 48 or 72 h incubation in FBS containing media.

### 2.4. Inhibitor Incubation

H2227, H446, and H196 cells were co-cultured with Entinostat (HY-12163, MCE, Monmouth Junction, NJ, USA) or 10058F4 (SC6650, Beyotime, Shanghai, China) for 48 h and collected for the following studies. The H2227 and H446 cells transfected with MYC-OE plasmid for 48 h were incubated with RGFP966 (HY-13909, MCE, Monmouth Junction, NJ, USA). After 48 h, the cells were collected for flow cytometry analysis.

### 2.5. Cell Proliferation Analysis

SCLC cells (5 × 10^3^) were seeded into 96-well plates treated with different concentrations of Entinostat (1 μM, 2 μM, 4 μM, 8 μM, 16 μM, and 32 μM). After 48 h, the numbers of live cells were detected by Cell Counting Kit-8 (CCK8) (C0038, Beyotime, Shanghai, China). Absorbance at 450 nm was measured. Cell viability = (OD_sample_ − OD_spontaneous_)/(OD_control_ − OD_spontaneous_) × 100%.

### 2.6. Cell Migration Analysis

SCLC cells (5 × 10^4^) were seeded into the chamber of BD BioCoat Matrigel coated plates (354480, BD, Franklin Lakes, NJ, USA). After 24 h, migrated cells were recorded after staining with crystal violet.

### 2.7. Western Blotting

Western blotting was conducted as described previously [17]. The parental and 72 h-transfected cells were lysed in RIPA buffer containing 1 mM phenylmethylsulphonyl fluoride. The supernatant was collected after the centrifuge at 15,000× *g* for 15 min at 4 °C and quantified using Enhanced BCA Protein Assay Kit (P0010, Beyotime, Shanghai, China). Equal amounts of protein were fractionated by sodium dodecyl sulfate polyacrylamide gel electrophoresis (SDS-PAGE), and transferred to polyvinylidene fluoride membranes (Millipore, Billerica, MA, USA), blocked with skimmed milk and then incubated overnight at 4 °C with different primary antibodies in buffer containing 5% skimmed milk. Membranes were washed with TBS containing 0.05% Tween-20 three times, incubated with a secondary antibody for 1 h at room temperature, and then washed again three times. The blotting was examined using chemiluminescence (P0018AM, Beyotime, Shanghai, China) and analyzed with Image J software (version 1.8.0). Antibodies against GAPDH (AP0066, bioworld, MN, USA), c-Myc (WL01781, Wanleibio, Shenyang, China; 10828-1-AP, Proteintech, Chicago, IL, USA), NeuroD1 (4373S, CST, Boston, MA, USA), YAP1 (14074S, CST, Boston, MA, USA), ASCL1 (ab74065, Abcam, Cambridge, UK), HDAC1 (WL01297, Wanleibio, Shenyang, China), HDAC2 (WL03149, Wanleibio, Shenyang, China), HDAC3 (WL02946, Wanleibio, Shenyang, China), and horseradish peroxidase-conjugated goat anti-rabbit IgG (RM3002, Ray Antibody Biotech, Beijing, China) were used.

### 2.8. Immunofluorescence Analysis

The cells were seeded in 24-well plates. After overnight incubation, the cells were washed with PBS and fixed with 4% paraformaldehyde for 20 min. After being washed in PBS, the cells were permeabilized with 0.1% Triton X-100 for 10 min and blocked with 5% bovine serum albumin. After 1 h incubation the cells were stained with rabbit anti-human c-Myc (10828-1-AP, Proteintech, Chicago, IL, USA, 1:500) at 4 °C overnight, followed by being incubated with fluorescein (FITC)-conjugated goat anti-rabbit IgG (SA00003-2, Proteintech, Chicago, IL, USA, 1:100) for 1 h at room temperature. After being washed, the cells were counterstained with antifade mounting medium with DAPI (P0131, Beyotime, Shanghai, China). The images of the cells were taken under the fluorescence microscope.

### 2.9. Flow Cytometry Analysis

The cultured cells were harvested, washed twice with PBS containing 2% FBS, and stained with PE-labelled mouse anti-human MICA/MICB (320906) or PE-labelled mouse IgG2a κ isotype ctrl antibody (400212, BioLegend, San Diego, CA, USA) for 30 min on ice in the dark followed by being washed twice with PBS containing 2% FBS. All stained cells were analyzed by FACSCanto II (BD, Franklin Lakes, NJ, USA). Live cells were carefully gated by forward and side scatter. Data were analyzed using FlowJo software (version 10).

### 2.10. RNA Isolation and Quantitative Real-Time PCR (qRT-PCR) Analysis

Total RNA was extracted using TRIzol Reagent (15596026, Invitrogen, Carlsbad, CA, USA) following the manufacturer’s instructions and reverse transcribed using First Strand cDNA Synthesis Kit (TOYOBO, Shanghai, China). Analyses of qRT-PCR were performed using SYBR qPCR Mix (QPS-201, TOYOBO, Shanghai, China) on a system (Cobas z480, Roche, Basel, Switzerland). The primers used for analysis were shown in Supplementary Table S1. The fold change of target mRNA expression was calculated based on the threshold cycle (Ct). *ACTB* was used as an internal control. Relative mRNA expression levels were analyzed using the 2^−ΔΔCt^ method.

### 2.11. Co-Immunoprecipitation (Co-IP) Assay

The lysates of the cultured H446 and H2227 cells were harvested and subjected to c-Myc immunoprecipitation using anti-c-Myc antibody (4 μg, 10828-1-AP, Proteintech, Chicago, IL, USA). Antibody-protein complexes were captured using 20 μL protein A + G sepharose beads (P2012, Beyotime, Shanghai, China). Immunoprecipitates were then analyzed by Western Blotting. The HRP conjugated light-chain specific mouse anti-rabbit IgG antibody (93702, CST, Boston, MA, USA) was used as secondary antibody, and rabbit IgG (3900S, CST, Boston, MA, USA) was used as a negative control.

### 2.12. Chromatin Immunoprecipitation (ChIP)-qPCR Assay

The predicted promoter sequence of *MICA* and *MICB* promoters were searched by UCSC Genome Browser (http://genome.ucsc.edu/; accessed on 4 February 2021) and Cistrome Data Browser (http://cistrome.org/db/#/; accessed on 4 February 2021). ChIP assay kit (P2078, Beyotime, Shanghai, China) was used for ChIP assay following the manufacturer’s instructions. Briefly, cells were fixed with 1% formaldehyde and quenched with 0.125 M glycine. Next, the cells were sonicated using an ultrasonic cell disruptor in the lysis buffer. DNA was immunoprecipitated with either control IgG (B900610, Proteintech, Chicago, IL, USA), H3K9ac (AF5611, Beyotime, Shanghai, China), H3K14ac (AF5614, Beyotime, Shanghai, China), H3K27ac (AF5620, Beyotime, Shanghai, China), HDAC3 (10255-1-AP, Proteintech, Chicago, IL, USA), or c-Myc (10828-1-AP, Proteintech, Chicago, IL, USA) primary antibody. RNA and protein were digested using RNase A (ST576, Beyotime, Shanghai, China) and Protein K (ST533, Beyotime, Shanghai, China), respectively. DNA were purified using DNA Purification Kit (D0033, Beyotime, Shanghai, China) followed by qPCR analysis. The qPCR primers are listed in Supplementary Table S2.

### 2.13. Cytotoxicity Assay

H2227, H196, or H446 cells (T) were seeded into 96-well plates (5 × 10^3^ cells per well) and cultured overnight. Next, the SCLC cell lines were co-cultured with NK-92MI cells (E) at different E/T ratio (2:1, 10:1, 50:1). After 4 h, the lysate of SCLC cells was tested using LDH Cytotoxicity Assay Kit (C0017, Beyotime, Shanghai, China). Absorbance at 490 nm was measured using a microplate reader (CLARIOstar, BMG LABTECH, Offenburg, Germany). Percent cell death was calculated as (OD_sample_ − OD_spontaneous_)/(OD_Max_ − OD_spontaneous_) × 100%.

### 2.14. Analysis of Differential Gene Expression from Cancer Cell Line Encyclopedia (CCLE) Dataset

Gene expression data of the SCLC cell lines were downloaded from CCLE (https://sites.broadinstitute.org/ccle; accessed on 9 March 2021). The correlation between *MYC* expression and *NKG2DL* expression was evaluated by Linear Regression test.

### 2.15. Kaplan-Meier Survival Analysis

To investigate the association between *MYC*, *MICA,* or *MICB* and the survival of patients, we downloaded the information related to the survival time of patients with lung cancer from Kaplan-Meier plotter (http://kmplot.com/analysis/; accessed on 8 January 2022).

### 2.16. Statistical Analysis

All experiments were performed in triplicate. Data were analyzed using GraphPad Prism software (version 8.0), and the results are presented as the mean ± SD. Comparisons between groups were conducted using analysis of unpaired *t* tests. Correlations between *MYC* and *NKG2DL* in SCLC cell lines were analyzed by Spearman’s rank test. *p* value < 0.05 was considered statistically significant.

## 3. Results

### 3.1. c-Myc, NKG2DL Expression and Susceptibility to NK Cell Killing of SCLC Cell Lines

**Figure 1:**
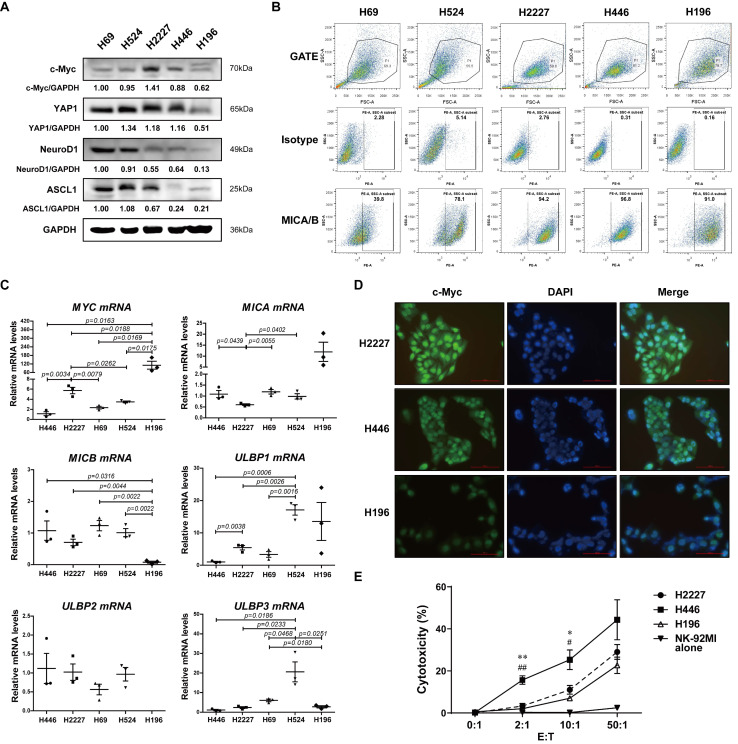
The expression of c-Myc and NKG2DL, and the susceptibility to NK-92MI cell killing in SCLC cell lines. (**A**) Western blotting spots of c-Myc, YAP1, NeuroD1, ASCL1, and GAPDH in H69, H524, H2227, H446, and H196 cells. (**B**) Flow cytometry analysis of MICA/B expression levels on the surface of the above cells. (**C**) qRT-PCR analysis of *MYC*, *MICA*, *MICB*, and *ULBP1-3* mRNA expression levels of the above cells. (**D**) Immunofluorescence staining of c-Myc in H2227, H446, and H196 cells (40×). Scale bar = 100 μm. (**E**) Cytotoxicity assay of the susceptibility of H2227, H446, and H196 cells to NK-92MI cell killing, respectively. Data are represented as mean ± SD (*n* = 3). *, #, *p* < 0.05; **, ##, *p* < 0.01. * *p*: H446 group vs. H2227 group; # *p*: H446 group vs. H196 group.

To explore the expression levels of c-Myc and NKG2DL of SCLC cell lines, we firstly detected the expression of c-Myc, key transcription regulators of SCLC-N and SCLC-Y (ASCL1, NeuroD1, and YAP1) [24] and major NKG2DL (MICA, MICB, ULBP1-3) [18] in H2227, H446, H69, H524, and H196 cells by Western Blotting, flow cytometry, and qRT-PCR. Consistent with the previous reports, H2227 and H446 cells were *MYC*-amplificated SCLC-N cells [25], both of which expressed c-Myc and neuroendocrine markers (Figure 1A and File S1). While the expression levels of MICA/B were exactly different: H446 and H196 cells expressed the highest level of MICA/B and H69 expressed the lowest level of MICA/B; H2227 and H524 cells expressed equivalent levels of MICA/B (Figure 1B). The qRT-PCR results showed that the expression level of *MYC* was higher in H2227 cells than in H446 cells, while the expression level of *MICA* was lower in H2227 cells than in H446 cells (Figure 1C). Further, the immunofluorescence assay and cytotoxicity assay were used to verify the c-Myc expression and susceptibility to NK cell killing of H2227, H446, and H196 cells. We found that compared to H446 and H196 cells, H2227 expressed a higher level of c-Myc and obtained a lower susceptibility to the killing by NK-92MI cells (Figure 1D,E). The above results indicated that c-Myc may be negatively correlated with NKG2DL expression and susceptibility to NK cell killing in SCLC-N cells.

### 3.2. c-Myc Inhibited Expression of NKG2DL and Susceptibility to NK Cell Killing in SCLC-N Cells

**Figure 2:**
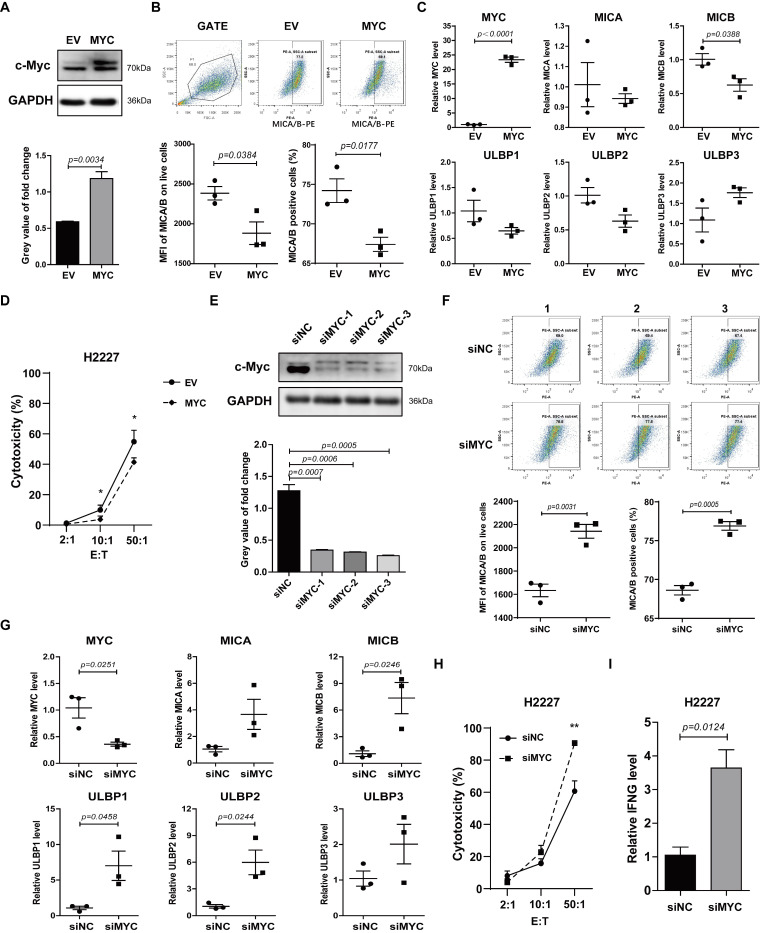
c-Myc was a negative regulator of NKG2DL in H2227 cells. H2227 cells were transfected with MYC-OE or *MYC* siRNAs (40 pmol/mL). (**A**,**E**) Western blotting analysis of the c-Myc expression levels in the transfected H2227 cells. (**B**,**F**) Flow cytometry analysis of MICA/B expression on the transfected H2227 cells. (**C**,**G**) qRT-PCR analysis of *MYC*, *MICA*, *MICB*, and *ULBP1-3* mRNA expression levels in H2227 cells after transfected with MYC-OE or *MYC* siRNA-3, respectively. (**D**,**H**) LDH analysis of the susceptibility of transfected H2227 cells to NK-92MI cell killing. (**I**) qRT-PCR analysis of *IFNG* mRNA expression levels in the transfected H2227 cells. Data are represented as mean ± SD (*n* = 3). *, *p* < 0.05; **, *p* < 0.01.

To verify whether c-Myc was involved in regulating the expression of NKG2DL in SCLC-N cells, we constructed the overexpression plasmid containing the CDS of human *MYC* gene and named it MYC-OE. H2227 and H446 cells were transfected with MYC-OE and the overexpression of c-Myc in H2227 cells was determined by western blotting (Figure 2A and Figure S1A). Further, flow cytometry analysis showed that the Median Fluorescence Intensity (MFI) of MICA/B and the percentage of MICA/B^+^ cells were lower in MYC-OE-transfected H2227 cells than in empty vector (EV)-transfected cells (Figure 2B). The results of qRT-PCR also showed that as *MYC* was up-regulated, the expression level of *MICB* in H2227 cells was down-regulated (Figure 2C). The cytotoxicity assay revealed that the susceptibility of H2227 cells to the killing by NK-92MI cells was decreased after MYC-OE transfection (Figure 2D).

Next, we transfected the human *MYC* siRNA (siMYC) into H2227 cells to silence c-Myc. As a result, siMYC transfection silenced the expression of c-Myc (Figure 2E), up-regulated the expression of MICA/B protein (Figure 2F), and *MICB*, *ULBP1*, and *ULBP2* mRNA (Figure 2G), enhanced the susceptibility to the killing by NK-92MI cells (Figure 2H) and the expression levels of *IFNG* in NK-92MI cells (Figure 2I). Similar results were obtained from H446 cells, in which c-Myc expression was up-regulated and silenced, respectively (Figures S1B and S2). The incubation of 10058F4 (the inhibitor of c-Myc) with SCLC cells obviously caused the co-cultured NK cells to aggregate into larger tumor spheres (Figure S3) which represented higher killing ability [27,28]. These data indicated a potential role for c-Myc in suppressing the expression of NKG2DL and anti-tumor immune response of NK cells in SCLC-N.

### 3.3. HDACs Involved in the Modulation of NKG2DL Expression in SCLC-N Cells

**Figure 3:**
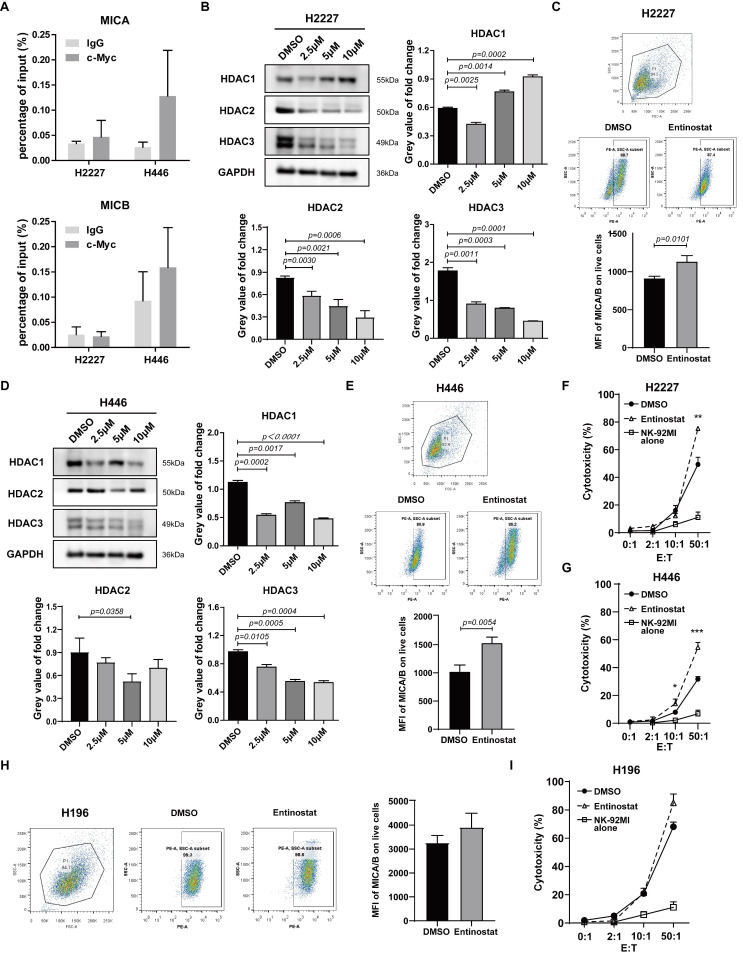
HDAC inhibitor modulated NKG2DL expression and susceptibility to NK cell killing of SCLC-N cells. (**A**) ChIP-qPCR analysis of c-Myc enrichment at *MICA* and *MICB* promoters in H2227 and H446 cells. (**B**,**D**) Western blotting analysis of HDAC1, HDAC2, and HDAC3 in H2227 and H446 cells treated with or without Entinostat (2.5 μM, 5 μM, and 10 μM). (**C**,**E**,**H**) Flow cytometry analysis of MICA/B expression levels on H2227, H446, and H196 cells treated with or without Entinostat (10 μM). (**F**,**G**,**I**) LDH analysis of the susceptibility to NK-92MI cell killing of H2227, H446, and H196 cells treated with or without Entinostat (10 μM). Data are represented as mean ± SD (*n* = 3). * *p* < 0.05, ** *p* < 0.01, *** *p* < 0.001 vs. DMSO group.

To explore the mechanism of c-Myc inhibiting MICA/B in SCLC, we used ChIP-qPCR assay to perform whether c-Myc directly binds to the promoter region of *MICA* or *MICB.* The data showed that c-Myc may not directly bind to the promoter of *MICA* or *MICB* in H2227 or H446 cells (Figure 3A). Recently, NKG2DL on SCLC-A cells was reported to be modulated by HDAC [18], which could interact with c-Myc to regulate gene expression [29]. Therefore, we speculated that c-Myc might regulate the expression of NKG2DL through HDACs in SCLC-N. Here, we found that the inhibitor of class I HDACs, the highly-expressed HDACs in the locally advanced, dedifferentiated, and strongly proliferating tumors [30], Entinostat, could inhibit the expression of HDAC1, HDAC2, and HDAC3, the three most studied class Ⅰ HDACs that this article focused on, with a final concentration greater than 2.5 μM in H2227 cells (Figure 3B), and inhibit the proliferation and migration of H2227 cells (Figure S4). Furthermore, the results of Flow Cytometry showed that Entinostat could up-regulate the MFI of MICA/B on H2227 cells (Figure 3C). Similar results were obtained from H446 cells (Figure 3D,E and Figure S4). Further, the results of cytotoxicity assay showed that Entinostat distinctly increased the susceptibility of H2227 cells and H446 cells to the killing by NK-92MI cells (Figure 3F,G). Intriguingly, the MFI of MICA/B and the susceptibility to NK cell killing of H196 cells, the SCLC-Y cells, were not significantly altered by Entinostat treatment (Figure 3H,I). The above results indicated that the class Ⅰ HDAC inhibitor, Entinostat, could induce the expression of MICA/B and improve the killing by NK cells of H2227 and H446 cells.

### 3.4. c-Myc Regulated NKG2DL Expression on SCLC-N Cells through HDAC3

**Figure 4:**
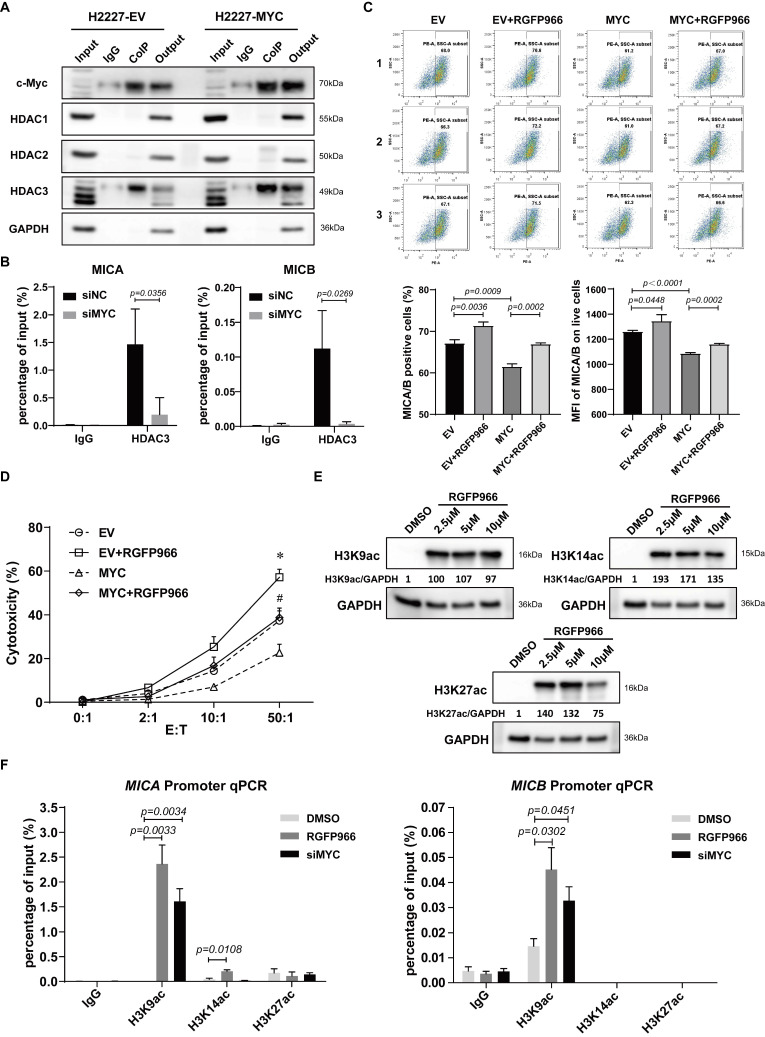
c-Myc modulated HDAC3 to deacetylate histone H3K9ac at *MICA* and *MICB* promoters. (**A**) Co-IP assay of the binding of c-Myc with HDAC1, HDAC2, or HDAC3 in H2227 cells transfected with or without MYC-OE. (**B**) ChIP-qPCR analysis of the binding of HDAC3 and *MICA/B* promoter in *MYC*-silencing H2227 cells. (**C**) Flow cytometry analysis of MICA/B expression in H2227 cells transfected with EV or MYC-OE followed the treatment of RGFP966 (10 μM) or not. (**D**) LDH analysis of the susceptibility to NK-92MI cell killing of H2227 cells transfected with EV or MYC-OE followed by the treatment of RGFP966 (10 μM) or not. (**E**) Western blotting analysis of H3K9ac, H3K14ac, and H3K27ac in H2227 cells treated with or without RGFP966 (2.5 μM, 5 μM, and 10 μM). (**F**) ChIP-qPCR analysis of H3K9ac, H3K14ac, and H3K27ac enrichment at *MICA* promoter and *MICB* promoter in H2227 cells treated with or without RGFP966 (10 μM) or transfected with siMYC. Data are represented as mean ± SD (*n* = 3). *, #, *p* < 0.05; * *p*: EV + RGFP966 group vs. EV group; # *p*: MYC + RGFP966 group vs. MYC group.

To clarify whether and which subtype of HDAC participated the modulation of c-Myc on NKG2DL expression in SCLC-N cells, we firstly detected the combination of c-Myc and HDAC1, HDAC2, or HDAC3 by Co-IP and found that only HDAC3 substantially interacted with c-Myc in H2227 and H446 cells (Figure 4A and Figure S5A). Further, the ChIP-qPCR analysis performed that HDAC3 could directly bind the promoter of *MICA* and *MICB* in control H2227 cells, and the binding was prevented by the transfection of siMYC (Figure 4B). Next, to confirm that c-Myc was indeed inhibiting the NKG2DL expression in SCLC-N cells through HDAC3, a highly selective HDAC3 inhibitor, RGFP966, was added to block the HDAC3 in H2227 cells after the transfection of MYC-OE. Since there is no effective inhibition of any other HDACs at concentrations of up to 15 μm [31,32], we chose the relatively high concentration, 10 μm, of RGFP966 to treat H2227 cells. The results of flow cytometry and LDH analysis showed that the percentage of MICA/B^+^ cells, MFI of MICA/B, and the susceptibility to NK-92MI cell killing of H2227 cells were decreased by the transfection of MYC-OE and recovered by RGFP966 (Figure 4C,D), suggesting that HDAC3 was the key molecule of c-Myc to suppress the expression of MICA/B and the susceptibility to NK cell killing of H2227 cells. Furthermore, we found RGFP966 could increase the acetylation of histone H3K9, H3K14, and H3K27 (Figure 4E), the possible sites of HDAC3 deacetylation [33,34,35], in H2227 cells. Importantly, the ChIP-qPCR assay confirmed that RGFP966 markedly increased the enrichment of H3K9ac, but not H3K14ac or H3K27ac, at the promoter of *MICA* and *MICB* in H2227 cells, which was recovered by the transfecting of siMYC (Figure 4F). The similar results were obtained from H446 cells (Figure S5). Collectively, these findings indicated that c-Myc suppressed the expression of NKG2DL in SCLC-N cells by recruiting HDAC3 to deacetylate H3K9ac at the promoter of *NKG2DL*.

### 3.5. Correlation between MYC and NKG2DL Expression in SCLC Cells and Lung Cancer Patients

**Figure 5:**
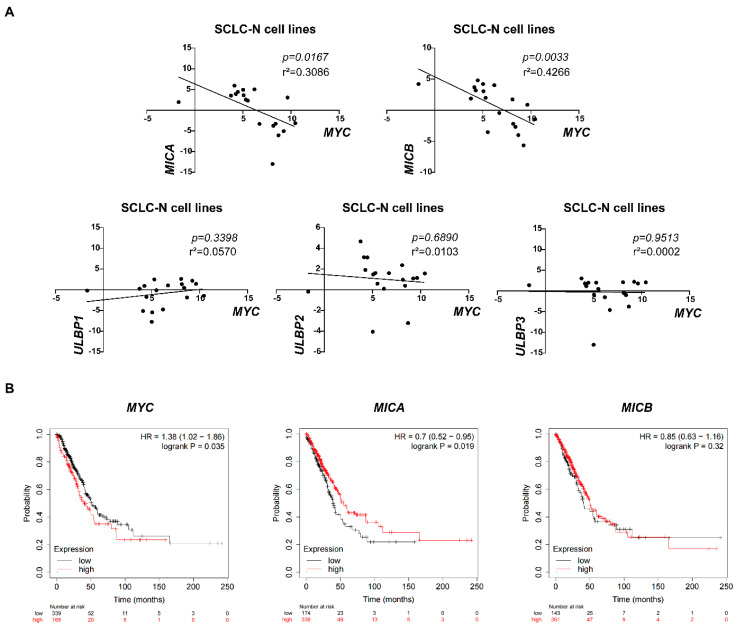
The negative correlation between *MYC* and *NKG2DL* expression in SCLC-N cells and lung cancer patients. (**A**) CCLE data analysis of the correlation between *MYC* and *MICA*, *MICB*, *ULBP1*, *ULBP2*, or *ULBP3* in SCLC-N cells lines, respectively (*n* = 18). (**B**) Kaplan-Meier survival curve of *MYC*, *MICA*, and *MICB* expression groups, respectively (*n* = 504).

In order to further determine the universality of the negative correlation between *MYC* and *NKG2DL* expression levels in SCLC cells, we analyzed the relationship between *MYC* and *NKG2DL* expression levels in online SCLC cell line data. CCLE data analysis showed that *MYC* mRNA expression levels were negatively associated with *MICA* and *MICB* mRNA expression levels in 18 SCLC-N cell lines [25] (*p* = 0.0167 and *p* = 0.0033, respectively) (Figure 5A, Table S3), but not in total 71 SCLC cell lines (Figure S6), indicating that the negative correlation between *MYC* and *NKG2DL* expression levels may only exist in SCLC-N. Due to the lack of studies on SCLC samples in the database, we analyzed the prognostic significance of *MYC* and *MICA/B* expression levels in lung cancer using Kaplan-Meier plotter to investigate the clinical significance of *MYC* and *NKG2DL*. We found that the patients with high *MYC* mRNA expression had worse OS (HR 1.38 [1.02–1.86], *p* = 0.0035), and the patients with high *MICA* mRNA expression had better OS (HR 0.7 [0.52–0.95], *p* = 0.019) (Figure 5B) which also suggested the negative correlation between *MYC* and *MICA* in lung cancer.

## 4. Discussion

NKG2DL has been considered as an activating signal on the surface of cancer cells to trigger the cytotoxicity cells including NK cells and CD8^+^ T cells and was the substantial mechanism of immune surveillance to initiate the immune response to find and eliminate NKG2DL^+^ tumor cells. In this study, we found that compared to the SCLC-Y cells, which was reported to express high level of inflammation gene and be infiltrated by more immune cells [8,36], SCLC-N cells expressed a lower level of NKG2DL, especially MICA/B (Figure 1B), which may be one of the reasons that SCLC-N was infiltrated with less immune cells [26]. Further, we found MICA and MICB, the most widely expressed NKG2DL, on SCLC-N cells were suppressed by *MYC* (Figure 2 and Figure S2), whose encoded protein, c-Myc, was higher expressed in SCLC-N cells than in other types of SCLC cells [8], indicating the key role of *MYC* in the immune characteristics of SCLC. In addition, previous studies reported that H2227 and H446 cells were SCLC-N cells [25] while H196 cells were SCLC-Y cells [24], which was consistent with our results that H2227 and H446 cells expressed higher level of c-Myc than H196 cells (Figure 1A,D). Since the structure and regulatory mechanism of ULBP family was quite different from MIC family [14,37,38,39], ULBPs may not be mainly regulated by *MYC*, which is consistent with the increased level of ULBP3 in both *MYC*-overexpressed and -silenced cells (Figure 2). Notably, among the five major NKG2DL, MICB was the main NKG2DL regulated by *MYC* in H2227 cells (Figure 2C), while MICA was the main one regulated by *MYC* in H446 cells (Figure S2C), indicating that the regulatory pathways of the same NKG2DL may be different in distinct tumor cells, which required to be further studied.

**Figure 6:**
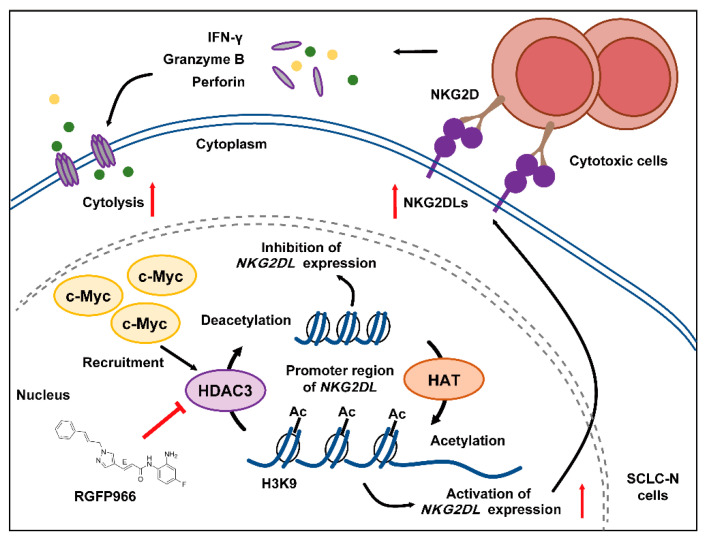
Schematic model of c-Myc regulating the expression of NKG2DL and the cytolysis in SCLC-N. c-Myc recruited and combined with HDAC3, which may facilitate the deacetylation of histone H3K9ac at the *NKG2DL* promoter region of SCLC-N cells, resulting in the decreased transcription level of *NKG2DL* and the inability to activate cytotoxic cells and the reduced cell lysis. HDAC3 inhibitor could reverse the decline of the cytotoxic cell killing effect by up-regulating the expression of NKG2DL.

Additionally, the study on the mechanism revealed that c-Myc recruited HDAC3 to deacetylate histone H3K9ac, which was bound to the promoter region of *NKG2DL* (Figure 4), thereby suppressing the transcription of *NKG2DL* in SCLC-N cells and inhibiting the cytotoxic effect of NK cells (Figure 2 and Figure 6). Consistently, Entinostat up-regulated NKG2DL expression and susceptibility to NK cell killing in SCLC-N, but not in SCLC-Y (Figure 3). Supporting our finding, rising evidence of RNA-seq showed the HDAC inhibitor sensitive gene signature of NEUROD1-subtype SCLC, but the immune system-enriched HDAC inhibitor resistant gene signature of SCLC-Y [40], which was consistent with the enrichment of immune signaling pathways in SCLC-Y [8,41,42]. However, it appeared that c-Myc did not up-regulate the expression of HDAC3 in SCLC-N, as the HDAC3 level in input was not up-regulated by overexpression of *MYC* (Figure 4A). The specific inhibitor of HDAC3, RGFP966, could recover the expression levels of NKG2DL and the susceptibility to the killing by NK cells that down-regulated by the overexpression of *MYC* (Figure 4), which was consistent with the reports that NKG2DL in SCLC-A subtype cells was suppressed by deacetylation [18]. Unexpectedly, we found that high concentration (10 µM) of RGFP966 did not significantly alter the acetylation levels of H3K9 and H3K14, but slightly down-regulated the level of H3K27 (Figure 4E), indicating that the compensatory mechanisms may be activated. Despite both the promoters of *MICA* and *MICB* could be regulated by HDAC3 (Figure 4B,F), only *MICB* mRNA was regulated by *MYC* (Figure 2C,G), indicating that *MICA* might be regulated by the unknown specific mechanism which needed further study. Moreover, SCLC-I tumors, which obtained the highest total immune infiltrate and cytolytic activity score [8], expressed higher level of NKG2DL. Whether the highly expressed NKG2DL are regulated by acetylation and the distinction in the acetylation level of NKG2DL in different types of SCLC is what we will focus on next. Considering for the increased susceptibility of SCLC-N cells to the killing of NK cells by both c-Myc inhibitor and HDAC inhibitor, we speculated that the application of HDAC inhibitor, especially HDAC3 inhibitor, might be an effective immunotherapy strategy targeting innate immunity for *MYC*-amplificated SCLC-N and could solve the problem that c-Myc is largely unstructured, falling in the category of ontrinsically disordered proteins [43] and is difficult to be targeted by small molecule inhibitors. Similarly, the effect of HDAC inhibitors and *MYC* on the cytotoxicity of NK cells is worthy of further study.

Here, it was found that c-Myc in SCLC-N cells could combined with HDAC3, but not HDAC1 or HDAC2 (Figure 4A), indicating that HDAC3 in SCLC-N cells might be combined with and recruited by c-Myc due to its special structure. In Non-Hodgkin B-cell lymphoma and acute myeloid leukemia, c-Myc was also reported to regulate the gene transcription by recruiting HDAC3 [44,45], while HDAC1/2 were most likely to be recruited to the promoter of *MYC* and regulated the transcription of *MYC* [46,47]. The above results were consistent with that HDAC1 and HDAC2 formed the functional complex with mSin3A, NuRD, and RCOR1/Co-REST, while HDAC3 formed a functional complex with N-CoR [48]. Due to the particularity of HDAC3, the mechanism of HDAC3 regulating NKG2DL transcription deserved further study. We found that HDAC3 may regulate the accessibility of the promoter and inhibit the transcription of *NKG2DL* by deacetylating histone H3K9ac and the treatment with HDAC3 specific inhibitors increased the acetylation level of H3K9 (Figure 4F) and up-regulated NKG2DL expression in SCLC-N cells (Figure 4C). Since the binding of HDAC3 and the promoters of *MICA/B* and the level of H3K9ac were prevented by the silencing of *MYC* (Figure 4B,F), it is further demonstrated the c-Myc/HDAC3 axis in the modulating of NKG2DL expression. This study proved the role and mechanism of HDAC3 in c-Myc-regulated SCLC-N immune characteristics, implying that HDAC3 may be a new target for the treatment of SCLC-N.

## 5. Conclusions

To date, evidence for c-Myc to participate in the regulation and mechanism of SCLC immune microenvironment and immune characteristics is still lacking. Based on our study, we confirmed that in *MYC*-amplificated SCLC cells, c-Myc was involved in regulating the expression of NKG2DL, the activating ligand for cytotoxic cells such as NK cells. The main mechanism might be that c-Myc recruited HDAC3 to deacetylate the histone H3K9ac, which bind to the promoter region of *NKG2DL*, thereby inhibiting the transcription of *NKG2DL*. Our findings suggested that the role of c-Myc in SCLC-N cells as a key modulator for immune characteristics and immunotherapy should be noted; compared with c-Myc, HDAC3 might be a better target for SCLC immunotherapy.

## Data Availability

Not applicable.

## Supplementary Materials

The following supporting information can be downloaded at: https://www.mdpi.com/article/10.3390/cancers14030457/s1, Figure S1: Expression levels of c-Myc in the transfected H2227 and H446 cells. Figure S2: c-Myc was a negative regulator of NKG2DL in H446 cells. Figure S3: The inhibition of c-Myc in SCLC-N cells enhanced the killing ability of NK-92MI cells in the co-culture system. Figure S4: Effect of Entinostat on proliferation and metastasis of H2227 and H446 cells. Figure S5: c-Myc modulated HDAC3 to deacetylate histone H3K9ac at *NKG2DL* promoter in H446 cells. Figure S6: Correlation between *MYC* and *NKG2DL* expression levels in SCLC cells. Table S1: Primers of specific genes used for qRT-PCR. Table S2: Primers used for ChIP-qPCR. Table S3: The expression levels of *MYC*, *MICA*, *MICB*, *ULBP1-3* mRNA in 18 SCLC-N cell lines (data form CCLE). File S1: Original Western blots figures.
