# Supplementary material for: c-Myc Targets HDAC3 to Suppress NKG2DL Expression and Innate Immune Response in N-Type SCLC through Histone Deacetylation
Source: Cancers (Basel). 2022 Jan 18;14(3):457. doi: 10.3390/cancers14030457 (PMC8833590; doi:10.3390/cancers14030457)

Western Blotting bands in Figure 1A

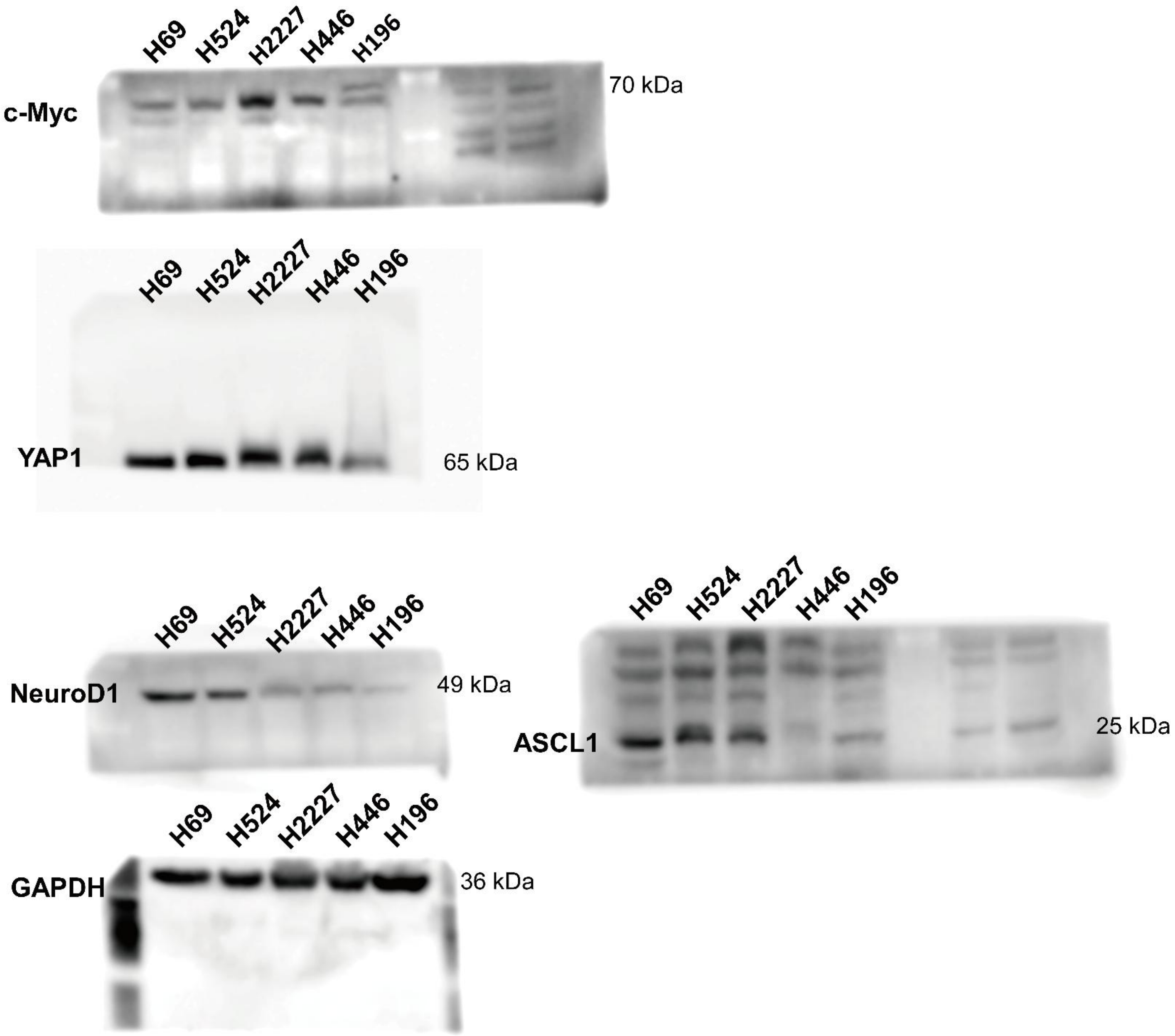

Western Blotting bands in Figure S1

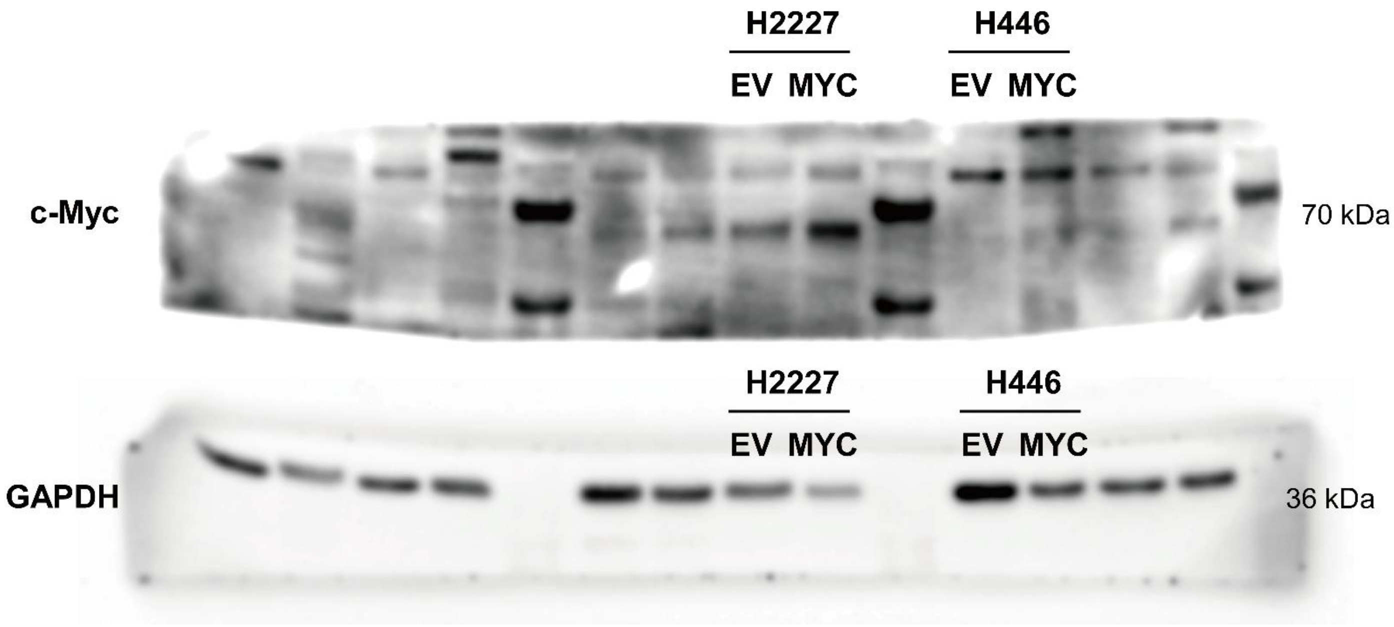

Western Blotting bands in Figure 2A and Figure S2A

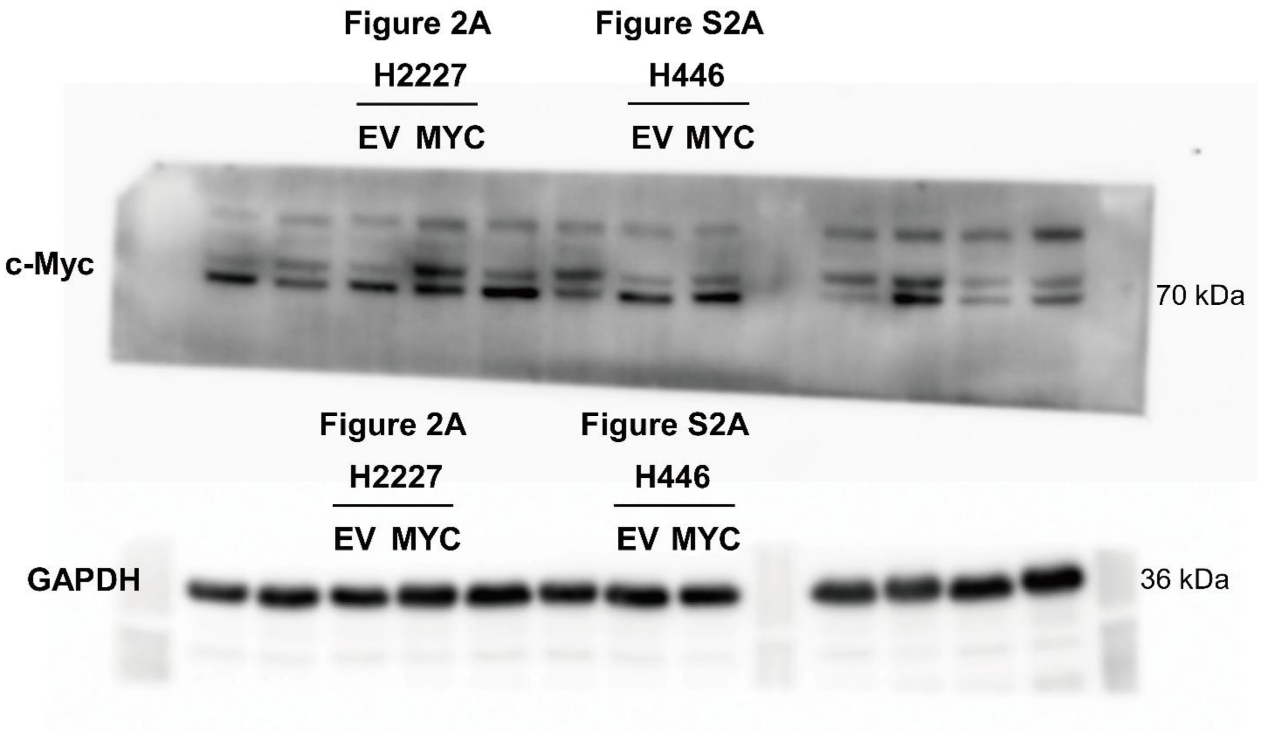

Western Blotting bands in Figure 2E and Figure S2E

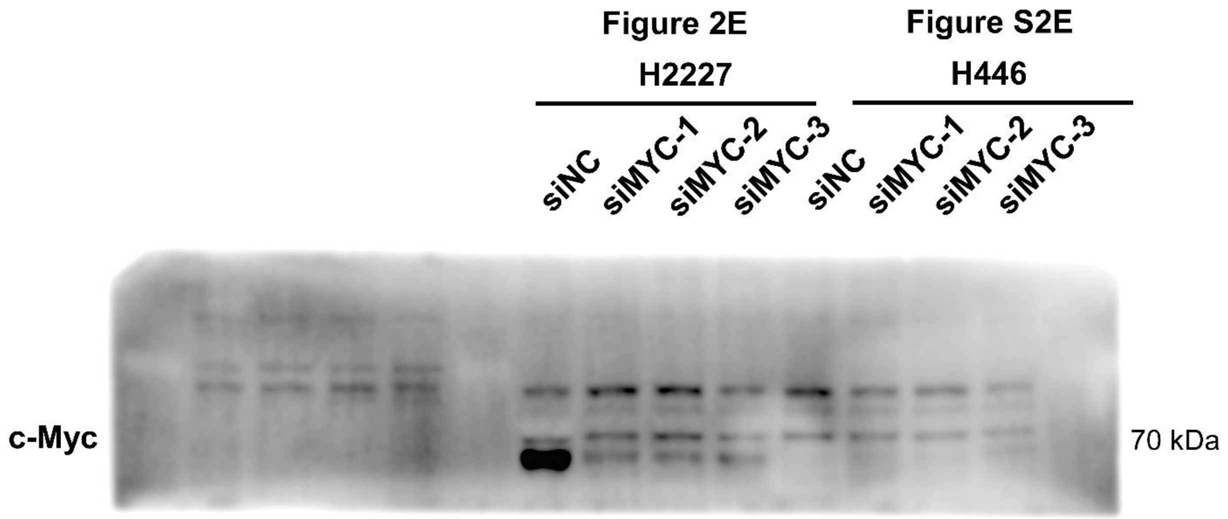

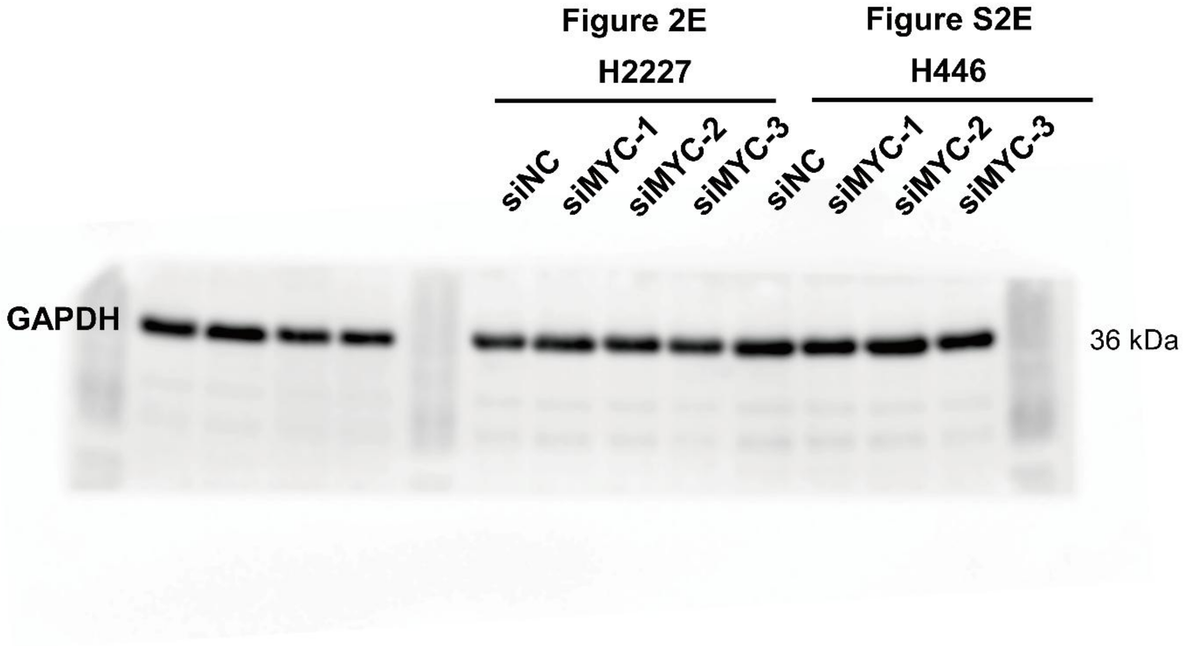

Western Blotting bands in Figure 3B and Figure 3D

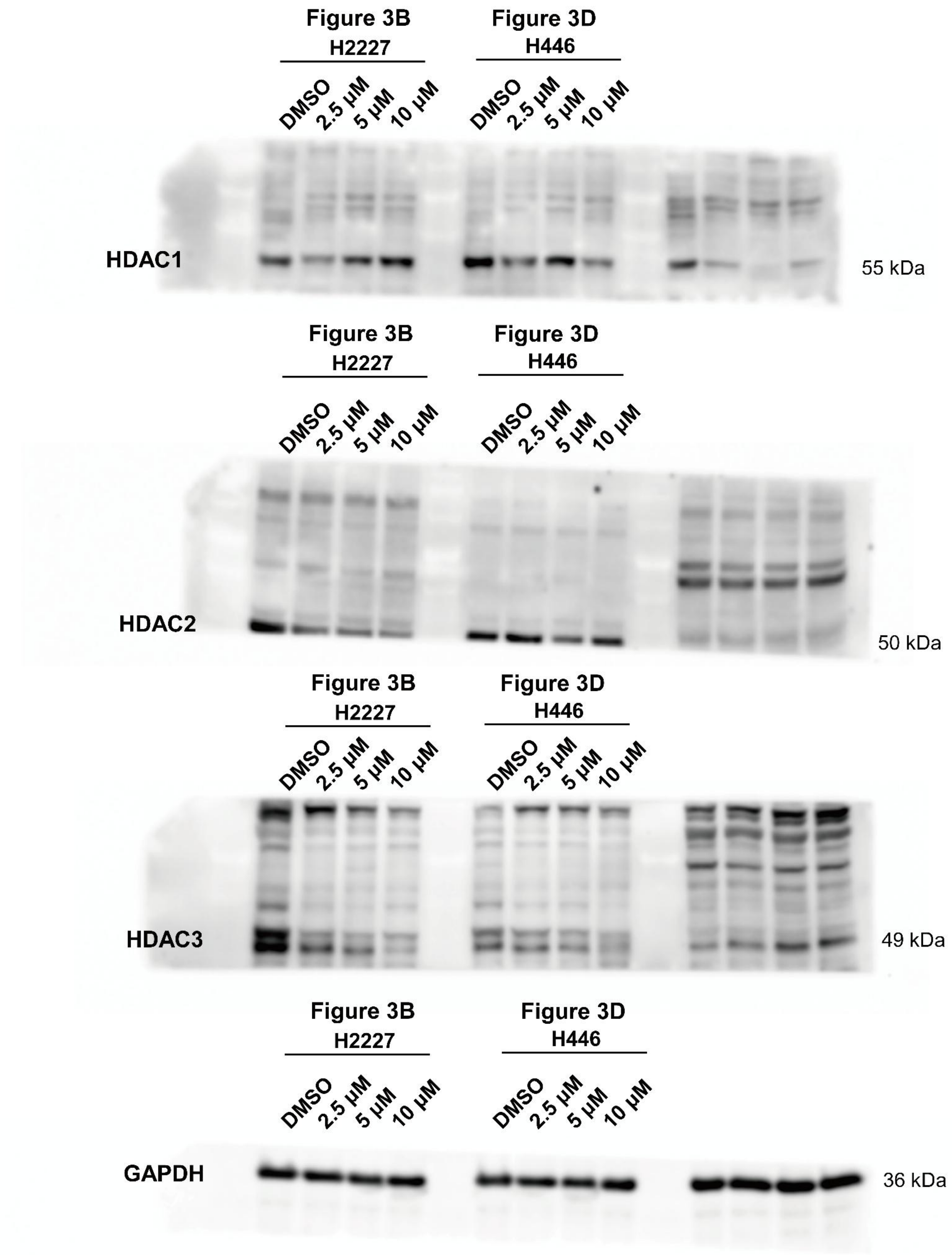

Western Blotting bands in Figure 4A

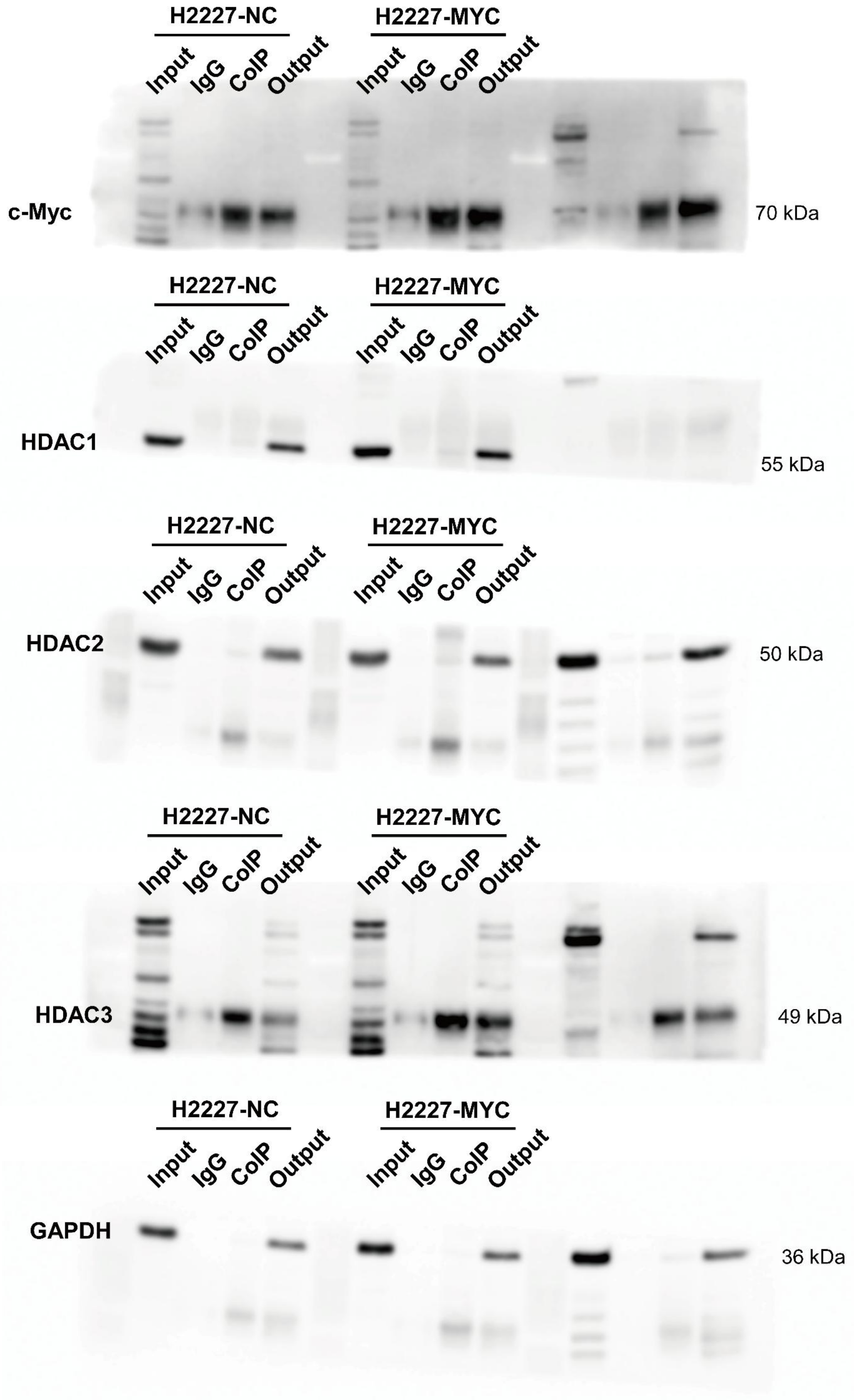

Western Blotting bands in Figure S5A

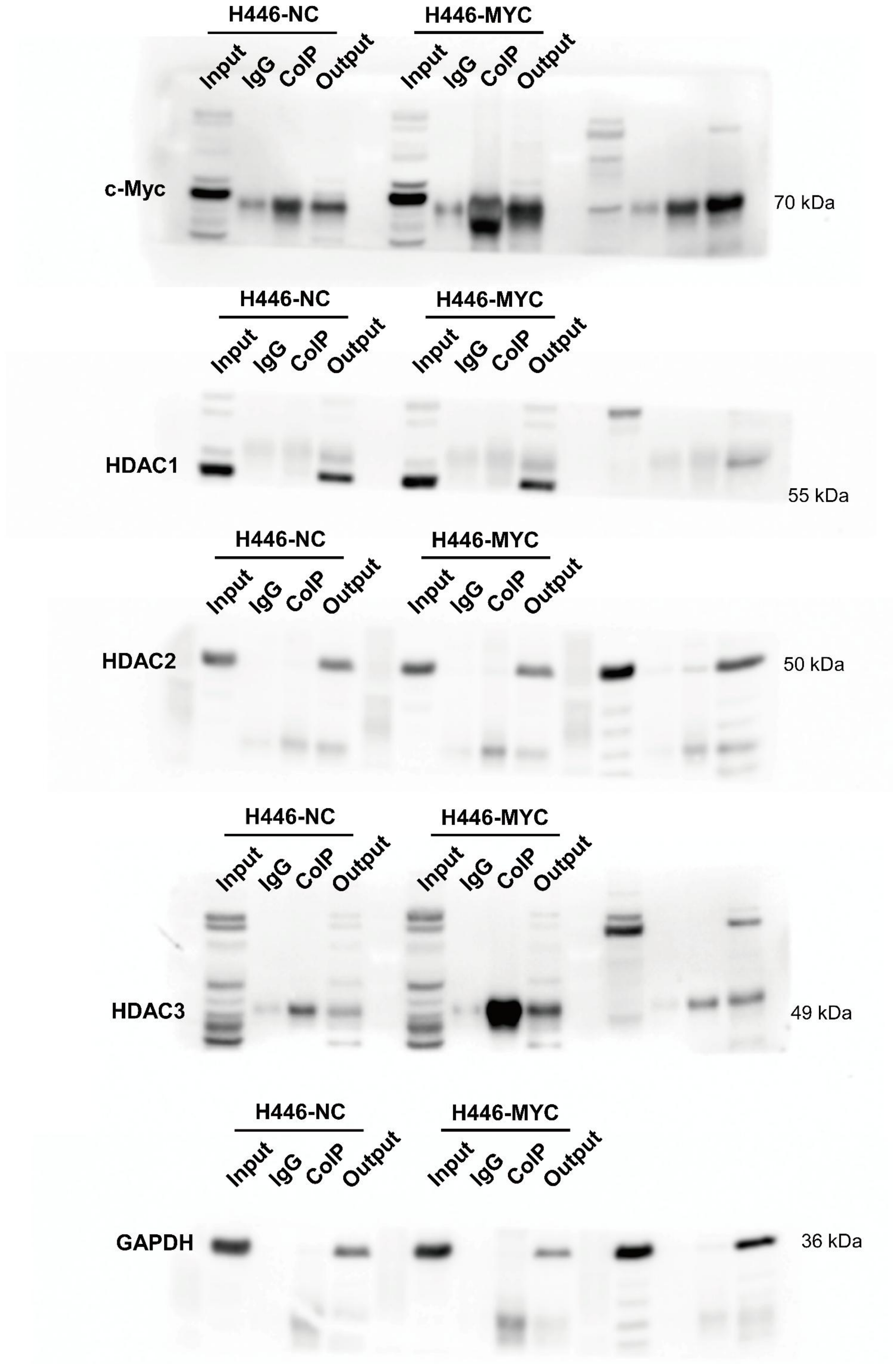

Western Blotting bands in Figure 4E

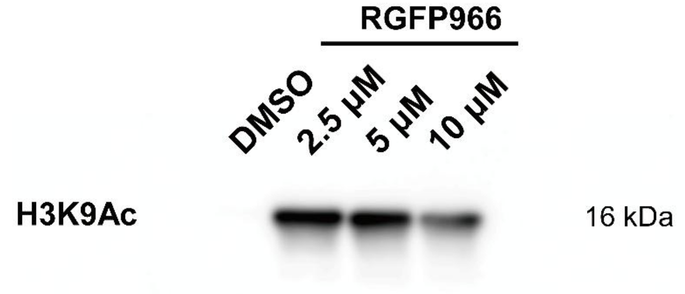

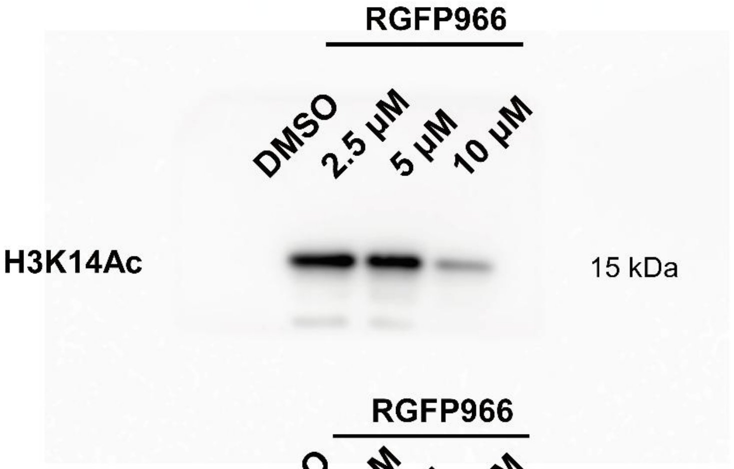

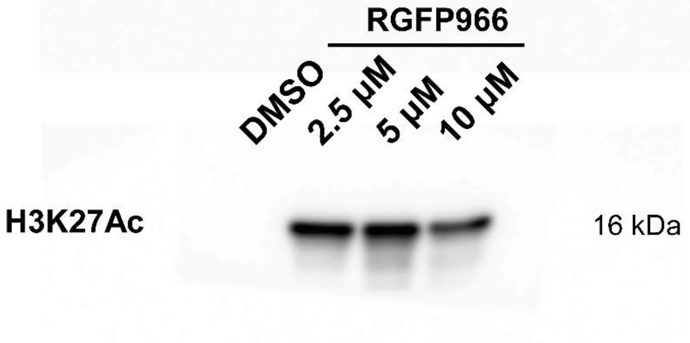

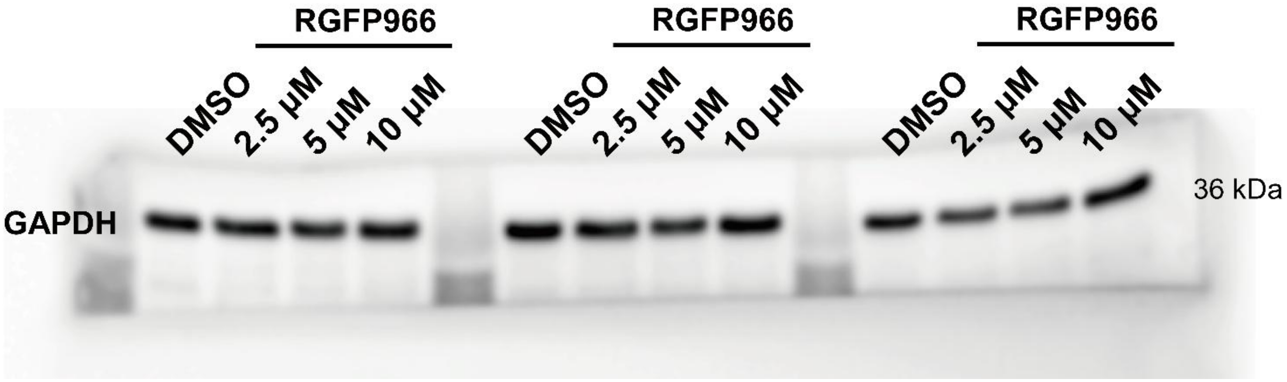

Western Blotting bands in Figure S5C

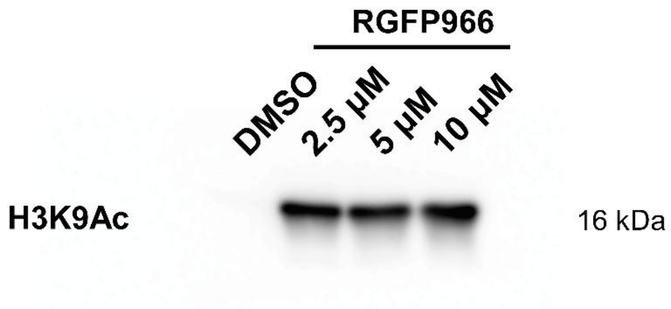

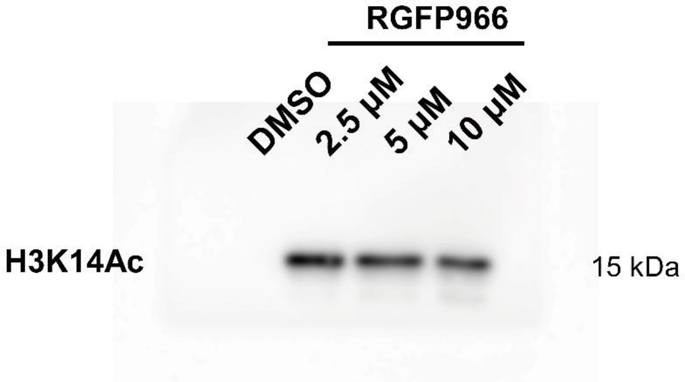

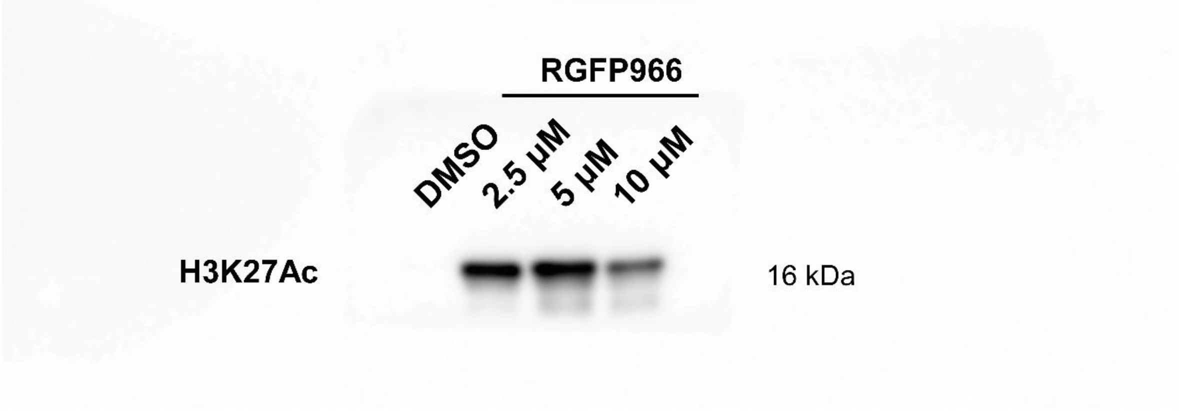

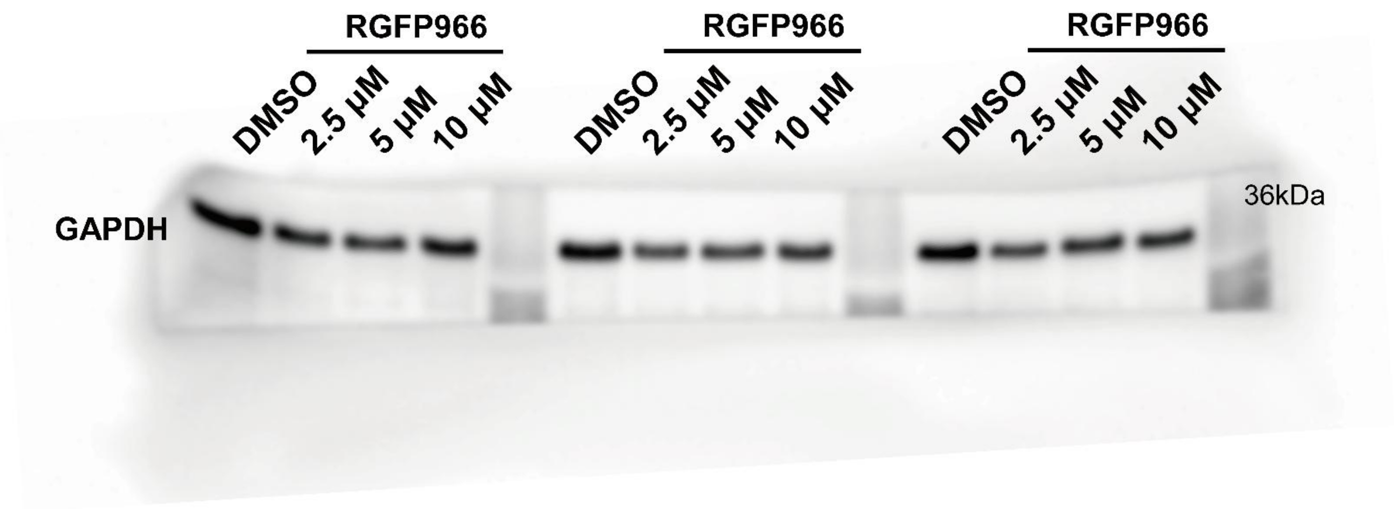

Supplement: Supplementary file 1 [file cancers-14-00457-s001.zip › cancers-1519271-supplementary/cancers-1519271 supplementary/cancers-1519271 File S1.pdf]
