# Supplementary material for: c-Myc Targets HDAC3 to Suppress NKG2DL Expression and Innate Immune Response in N-Type SCLC through Histone Deacetylation
Source: Cancers (Basel). 2022 Jan 18;14(3):457. doi: 10.3390/cancers14030457 (PMC8833590; doi:10.3390/cancers14030457)
Supplement: Supplementary file 1 [file cancers-14-00457-s001.zip › cancers-1519271-supplementary/cancers-1519271 supplementary/cancers-1519271-Figure S1-S6 and Table S1-S3.pdf]

cancers

MDPI

c-Myc Targets HDAC3 to Suppress NKG2DL Expression and Innate Immune Response in N-Type SCLC through Histone Deacetylation

Peiyan Zhao [1], Xiaodan Sun [1], Hui Li [2], Yan Liu [2], Yanan Cui [3], Lin Tian [2] and Ying Cheng [2,3,*]

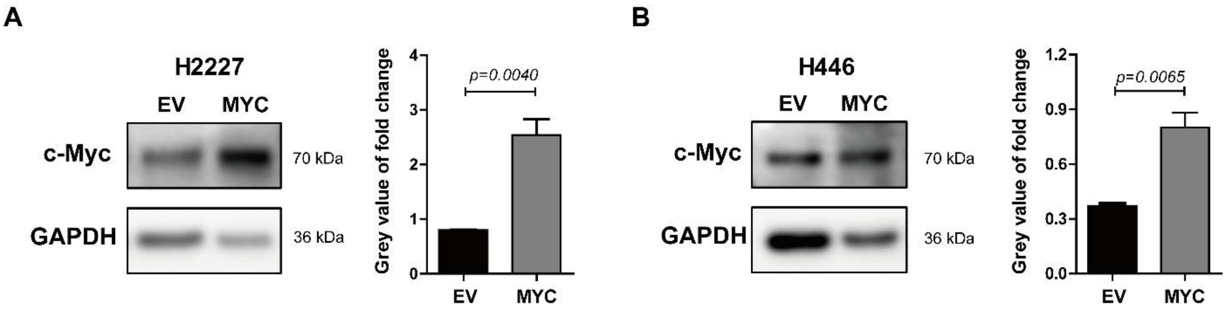

Figure S1. Expression levels of c-Myc in the transfected H2227 and H446 cells. H2227 cells and H446 cells were transfected with EV or MYC-OE plasmids. Western blotting analysis of c-Myc expression levels in the transfected H2227 cells (A) and H446 cells (B) using another c-Myc antibody. Data are represented as mean ± SD (n = 3).

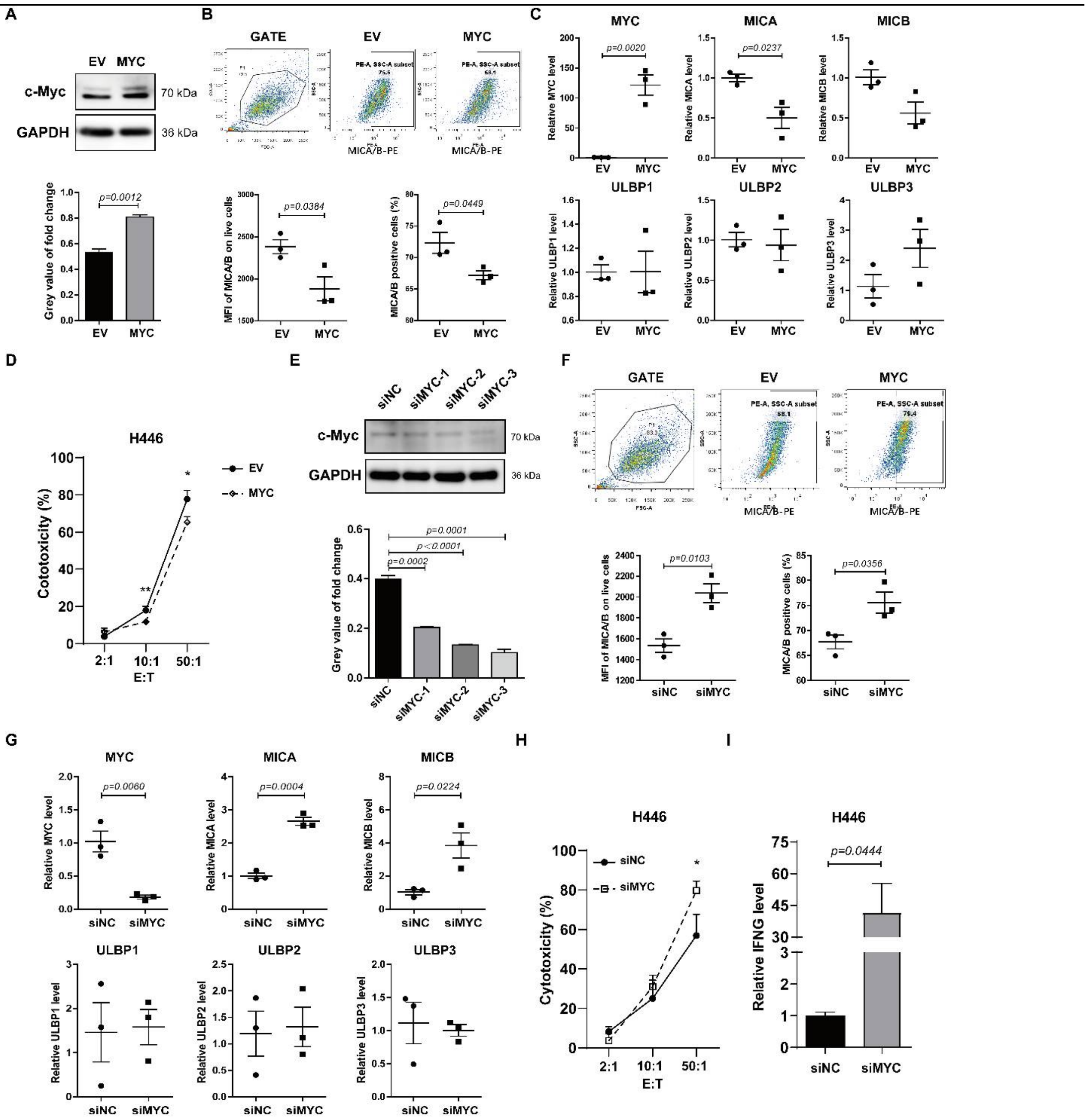

Figure S2. c-Myc was a negative regulator of NKG2DL in H446 cells. H446 cells were transfected with MYC-OE or MYC siRNAs (40 pmol/ml). (A, E) Western blotting analysis of the c-Myc expression levels in the transfected H446 cells. (B, F) Flow Cytometry analysis of MICA/B expression on the transfected H446 cells. (C, G) qRT-PCR analysis of *MYC*, *MICA*, *MICB*, and *ULBP1-3* mRNA expression levels in H446 cells after transfected with MYC-OE or MYC siRNA-3, respectively. (D, H) LDH analysis of the susceptibility of transfected H446 cells to NK-92MI cell killing. (I) qRT-PCR analysis of *IFNG* mRNA expression levels in the transfected H446 cells. Data are represented as mean ± SD (n = 3). *, p<0.05; **, p<0.01.

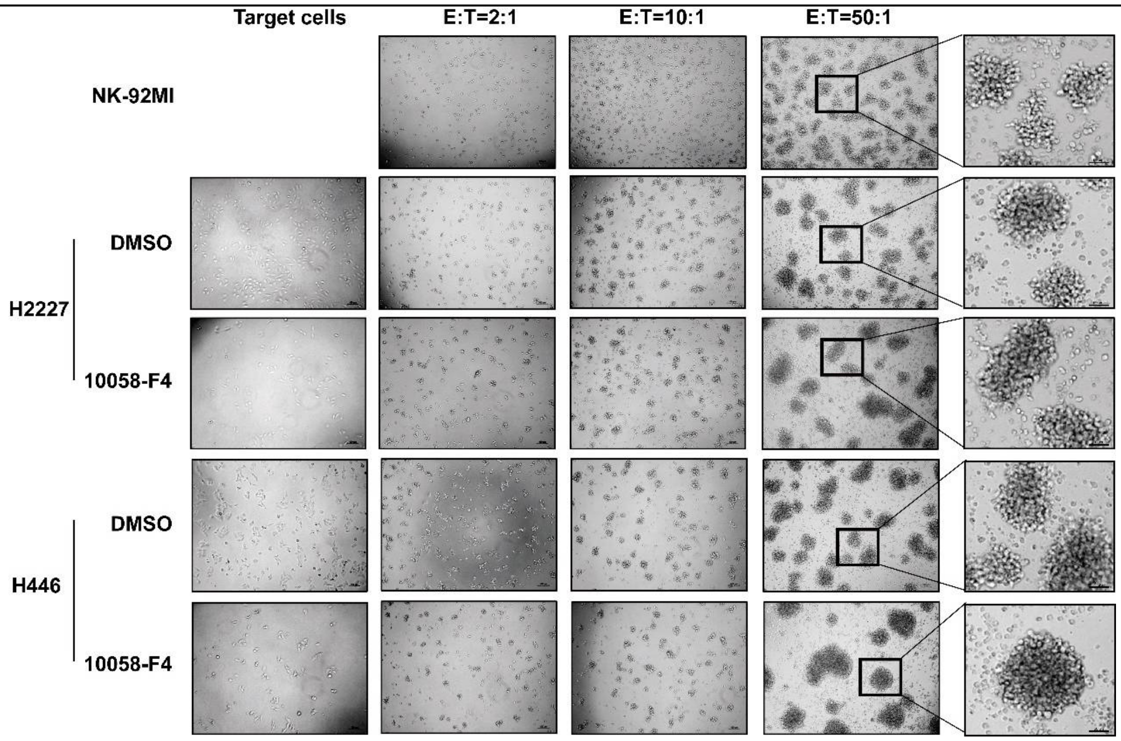

Figure S3. The inhibition of c-Myc in SCLC-N cells enhanced the killing ability of NK-92MI cells in the co-culture system. The cell clusters formed by NK-92MI cells co-cultured with H2227 or H446 cells with DMSO or 10058-F4 (50 μM) for 48 h were recorded. Scale bars, 100 μm and 50 μm.

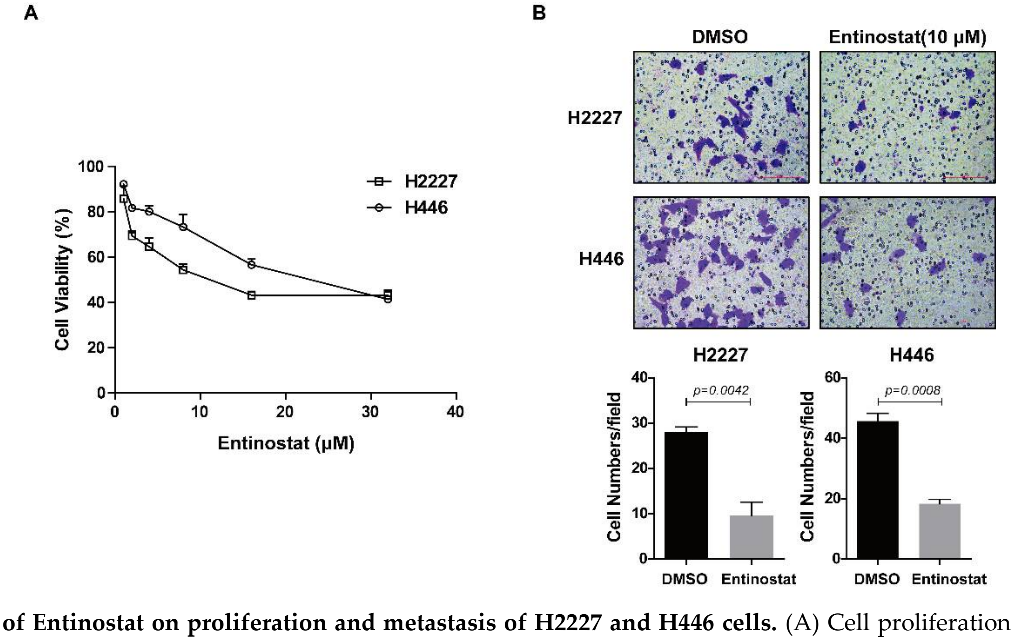

Figure S4. Effect of Entinostat on proliferation and metastasis of H2227 and H446 cells. (A) Cell proliferation analysis of H2227 and H446 cells treated with different concentrations of Entinostat for 48 h. (B) Transwell assay of H2227 and H446 cells treated with DMSO or Entinostat (10 μM) for 48 h. Scale bars, 100 μm. Data are represented as mean ± SD ($n = 3$).

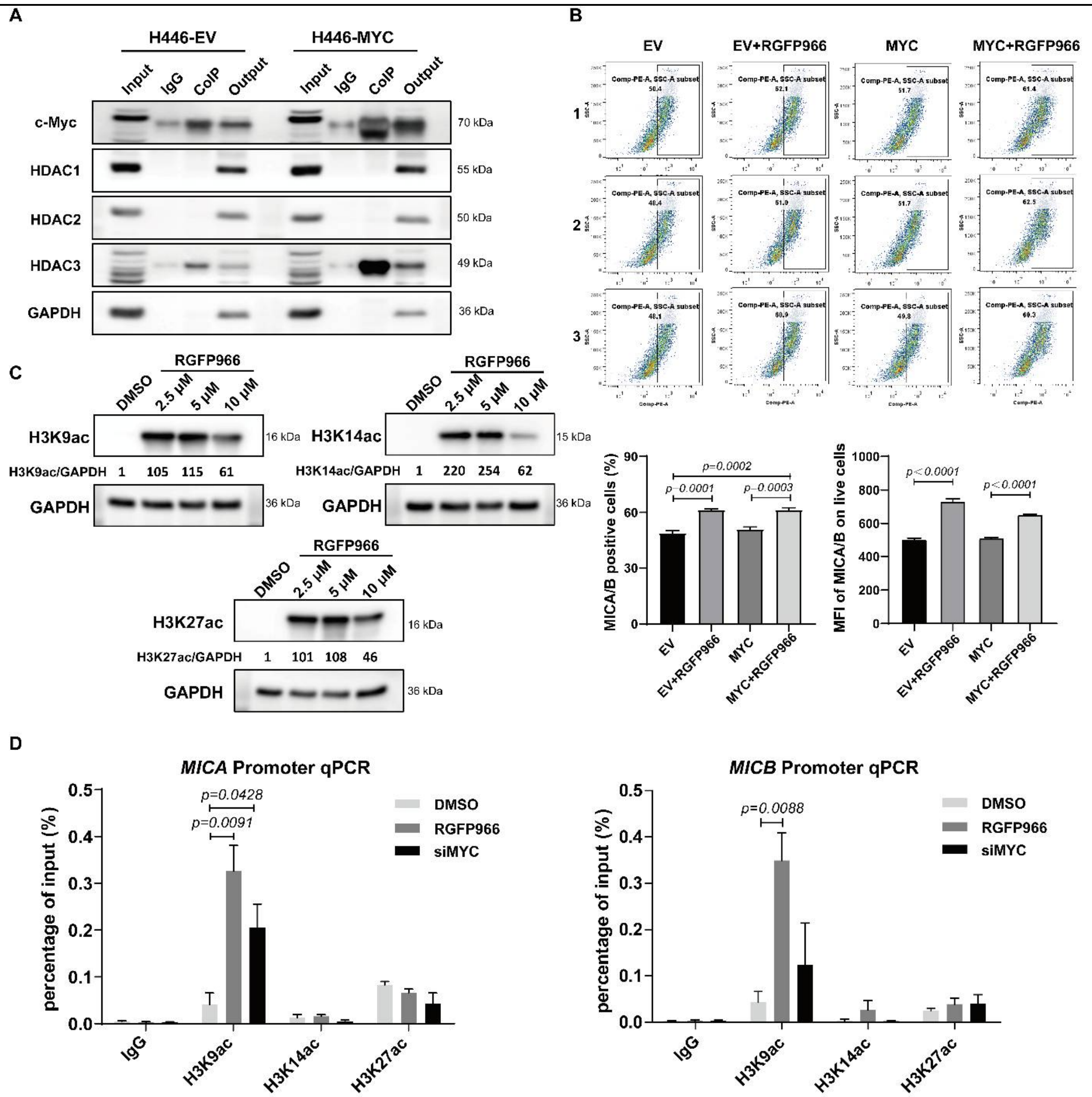

Figure S5. c-Myc modulated HDAC3 to deacetylate histone H3K9ac at *NKG2DL* promoter in H446 cells. (A) Co-IP assay of the binding of c-Myc with HDAC1, HDAC2 or HDAC3 in H446 cells transfected with or without MYC-OE. (B) Flow Cytometry analysis of MICA/B expression in H446 cells transfected with EV plasmid or MYC-OE followed the treatment of RGFP966 (10 μM) or not. (C) Western Blotting analysis of H3K9ac, H3K14ac and H3K27ac in H446 cells treated with or without RGFP966 (2.5 μM, 5 μM and 10 μM). (D) ChIP-qPCR analysis of H3K9ac, H3K14ac and H3K27ac enrichment at *MICA* promoter and *MICB* promoter in H446 cells treated with or without RGFP966 (10 μM), or transfected with siMYC. Data are represented as mean ± SD ($n = 3$).

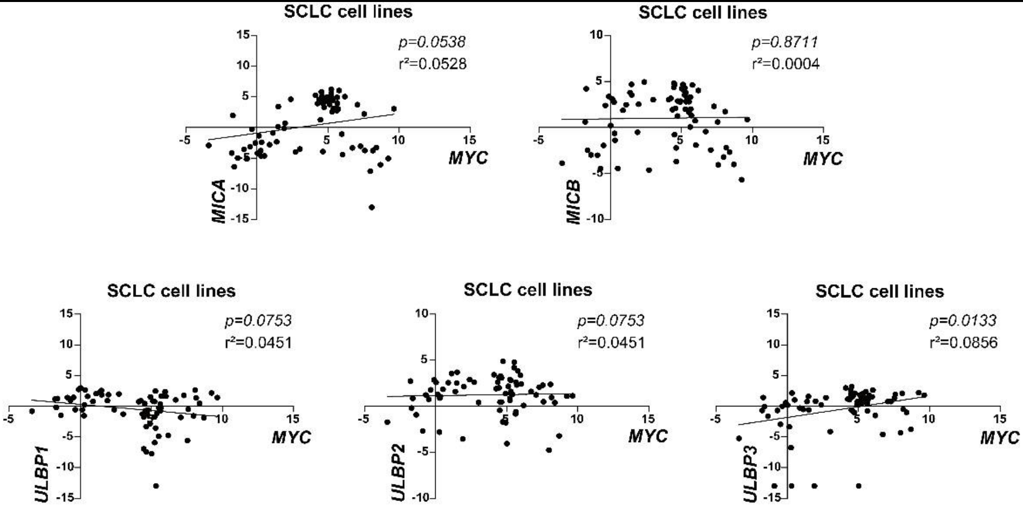

Figure S6. Correlation between *MYC* and *NKG2DL* expression levels in SCLC cells. CCLE data analysis of the correlation between *MYC* and *MICA, MICB, ULBP1, ULBP2* or *ULBP3* in SCLC cells lines, respectively ($n = 71$).

Table S1. Primers of specific genes used for qRT-PCR.

Target gene	Primer sequence (5'→3')	
	Forward	Reverse
MYC	TCAAGAGGTGCCACGTCTCC	TCTTGGCAGCAGGATAGTCCTT
MICA	GGCATCTTCCCTTTTGCAC	GGACAGCACCGTGAGGTTAT
MICB	GGTGCTTCAGAGTCAACGGACA	CAGGACCTGCAGGCTCACAA
ULBP1	TTCATTCTAGCTGGCAGATGAGG	TGGGCTTGCTAAGGCTTGAAC
ULBP2	ACTATGACTGTGGCAACAAGAC	CTGCCCATCGAAACTGAACTG
ULBP3	TCTATGGGTCACCTAGAAGAGC	TCCACTGGGTGTGAAATCCTC
ACTB	TGGCACCCAGCACAATGAA	AGTCATAGTCCGCCTAGAAGCA

Table S2. Primers used for ChIP-qPCR.

Target gene	Primer sequence (5'→3')	
	Forward	Reverse
MICA	GATGGGTTCCAAGGCAGAGG	ATCTCCCCAGGGTTGCAGTA
MICB	TCACGGGTTTCATTCAGTTGG	ACTCCAGACAGCACACCTGAG

Table S3. The expression levels of *MYC, MICA, MICB, ULBP1-3* mRNA in 18 SCLC-N cell lines (data form CCLE).

	MYC	***MICA***	***MICB***	***ULBP1***	***ULBP2***	***ULBP3***
H2227	-1.68128509	1.956630152	4.206016911	-0.191825298	-0.183816807	1.400750474
H446	6.718703855	-3.317171681	-0.442702485	1.025634344	1.60018674	-4.594361981
H524	9.240763387	-5.045750431	-5.656707999	2.16151097	1.086043712	2.200817206
H2171	8.080235905	-13	1.74731039	2.608113453	2.378196146	2.107764462
H1651	4.259441	3.910619493	3.177068339	0.900322256	1.915632876	1.176037521
COLO320	10.44885056	-3.136151471	-1.436769042	-1.45561364	1.577195417	2.099938309
COLO800	4.118404565	5.947513968	3.711908488	-5.130078917	3.148607689	1.820307435
COV644	3.764357338	3.53669519	1.87972514	0.24153997	4.664336167	3.028270654
HCC1438	4.470992316	4.425615202	4.813904561	-1.737369753	3.114931215	1.986453677
H1869	6.196604077	5.032925337	4.018582578	-4.763189589	0.097060739	-1.509126972
H1930	5.291461189	2.555828234	1.995712902	2.488134952	1.610462013	2.000390864
H2110	5.025690604	4.932153373	4.228371742	-7.732670079	-4.053445721	-13
H2452	5.069796046	3.623189941	3.056800422	-5.46512425	1.473486896	-0.921554836
RH30	5.519092104	2.324349641	-3.522921141	-0.148739538	0.583427829	0.513018765
CORL279	8.156898263	-3.768058881	-2.189982689	1.35130902	0.965858337	-0.493118132
DMS273	9.642028439	3.066532027	0.876224649	1.420112499	1.133416937	1.824385152
H82	8.699378327	-6.070202524	-4.003088156	-1.858640904	-3.22381057	-3.774503195
SCLC21H	8.394347551	-3.260359564	-2.673871056	0.45934894	0.387486491	-0.978466073
